# Quantitative morphological analysis framework of infant cranial sutures and fontanelles based on CT images

**DOI:** 10.1111/joa.14056

**Published:** 2024-05-09

**Authors:** Siyuan Chen, Svein Kleiven, Ingemar Thiblin, Xiaogai Li

**Affiliations:** ^1^ Division of Neuronic Engineering, Department of Biomedical Engineering and Health Systems KTH – Royal Institute of Technology Huddinge Sweden; ^2^ Forensic Medicine, Department of Surgical Sciences Uppsala University Uppsala Sweden

**Keywords:** infant suture, infant fontanelle, morphological variation, morphology analysis framework, statistical model

## Abstract

Characterizing the suture morphological variation is a crucial step to investigate the influence of sutures on infant head biomechanics. This study aimed to establish a comprehensive quantitative framework for accurately capturing the cranial suture and fontanelle morphologies in infants. A total of 69 CT scans of 2–4 month‐old infant heads were segmented to identify semilandmarks at the borders of cranial sutures and fontanelles. Morphological characteristics, including length, width, sinuosity index (SI), and surface area, were measured. For this, an automatic method was developed to determine the junction points between sutures and fontanelles, and thin‐plate‐spline (TPS) was utilized for area calculation. Different dimensionality reduction methods were compared, including nonlinear and linear principal component analysis (PCA), as well as deep‐learning‐based variational autoencoder (VAE). Finally, the significance of various covariates was analyzed, and regression analysis was performed to establish a statistical model relating morphological parameters with global parameters. This study successfully developed a quantitative morphological framework and demonstrate its application in quantifying morphologies of infant sutures and fontanelles, which were shown to significantly relate to global parameters of cranial size, suture SI, and surface area for infants aged 2–4 months. The developed framework proved to be reliable and applicable in extracting infant suture morphology features from CT scans. The demonstrated application highlighted its potential to provide valuable insights into the morphologies of infant cranial sutures and fontanelles, aiding in the diagnosis of suture‐related skull fractures. Infant suture, Infant fontanelle, Morphological variation, Morphology analysis framework, Statistical model.

## Graphical abstract

By utilizing 69 CT images of infants aged 2–4 months, the developed framework identifies and quantifies morphological variations in infant sutures and fontanelles. The collected data on infant sutures and fontanelles can serve as a reference for pediatricians and other researchers in this field.

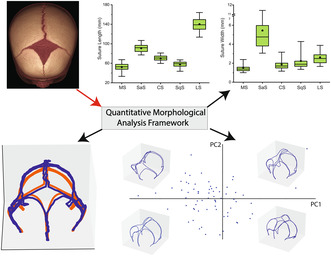

## 1 INTRODUCTION

Infant skull fractures may occur in falls from a low height such as falls from a bed (Kokulu et al., 2021). Skull fractures are also present in suspected abusive head trauma (AHT), posing a substantial challenge in forensic diagnosis (Isaac et al., 2023; Li et al., 2019; Reece & Sege, 2000; Sidpra et al., 2021). Infant skull fractures are often suture‐related, sometimes crossing sutures (Kriss et al., 2021; Weber et al., 1984). Finite element (FE) head models are powerful numerical tools to study infant head injury mechanics and skull fractures allowing the incorporation of sutures and fontanelles (Coats et al., 2007; He et al., 2020; Li et al., 2017, 2019; Roth et al., 2008, 2010). Such models could provide subject‐specific information adding diagnosis of AHT. However, quite often the diagnosis of AHT needs to refer to epidemiological studies to infer the likelihood of skull fracture for a specific case based on epidemiological data. In this case, it is crucial to understand the morphological variation of suture and fontanelle among infants and its influence on skull fractures, especially since suture patterns are highly individualistic (Sekharan, 1985) and the morphology is complicated.

Besides being closely linked to the biomechanics of infant skull fractures, sutures and fontanelles, as an integral part of the craniofacial skeleton, play a crucial role in normal skull growth as the brain grows (Cunningham & Heike, 2007). Abnormal suture development is highly related to skull dysmorphology (Persing, 2008). Brian et al. (Showalter et al., 2012) investigated the influence of frontosphenoidal suture synostosis on skull dysmorphology. Synostosis of the squamosal suture (SqS) can result in significant craniofacial dysmorphism (Smartt Jr et al., 2012). Therefore, the individual variation of infant cranial sutures and fontanelles should be statistically investigated, and most importantly, a systematic framework must be established for quantitatively analyzing infant cranial suture morphology.

Morphological studies related to human head have been performed in the past based on computed tomography (CT) and magnetic resonance imaging (MRI). Neubauer et al. (Neubauer et al., 2009) investigated endocranial bony structure shape changes during human postnatal ontogeny based on 108 CT scans of different ages from newborns to adults. Loyd et al. (Loyd et al., 2010) analyzed three‐dimensional pediatric head and skull contours data based on 185 clinical CT scans. Li et al. (Li et al., 2015) developed a statistical head model for children aged 0–3 years based on 56 head CT scans, followed by the development of a statistical head model for 3–10 year‐old children based on 42 head CT scans (Li et al., 2022). Wei et al. (Wei et al., 2022) developed skull and scalp statistical geometry models among the adolescent and young adult population based on 101 CT scans. Mercan et al. (Mercan et al., 2020) quantified the skull bone growth for the first 6 months of life in both normal and sagittal synostosis patients by using the PCA method. Mohtasebi et al. (Mohtasebi et al., 2021) modeled the development of the neonatal skull using 19 CT images. Passe et al. (Passe et al., 1997) investigated age and sex effects on brain morphology with MRI results of 43 normal subjects aged 24–82 years old. Although the aforementioned studies have enriched our understanding of the morphological differences and growth rules of the human head and skull, there is a scarcity of study that takes the detailed cranial suture and fontanelle variation into consideration.

Major developmental cranial sutures include the metopic, sagittal, coronal, lambdoid, and squamosal sutures. Idriz et al. (Idriz et al., 2015) described the normal anatomy of major pediatric sutures and common variants. To the best knowledge of the authors, no statistical shape model describing infant suture and fontanelle morphological variation has been developed and a quantitative framework has yet to be established for developmental sutures. Therefore, the present study aims to develop such a framework to analyze the morphology of infant cranial suture and fontanelle, and we demonstrate its application by quantitatively evaluating the morphology variation of major cranial sutures based on CT images for infants aged 2–4 months.

## 2 METHODS

**FIGURE 1:**
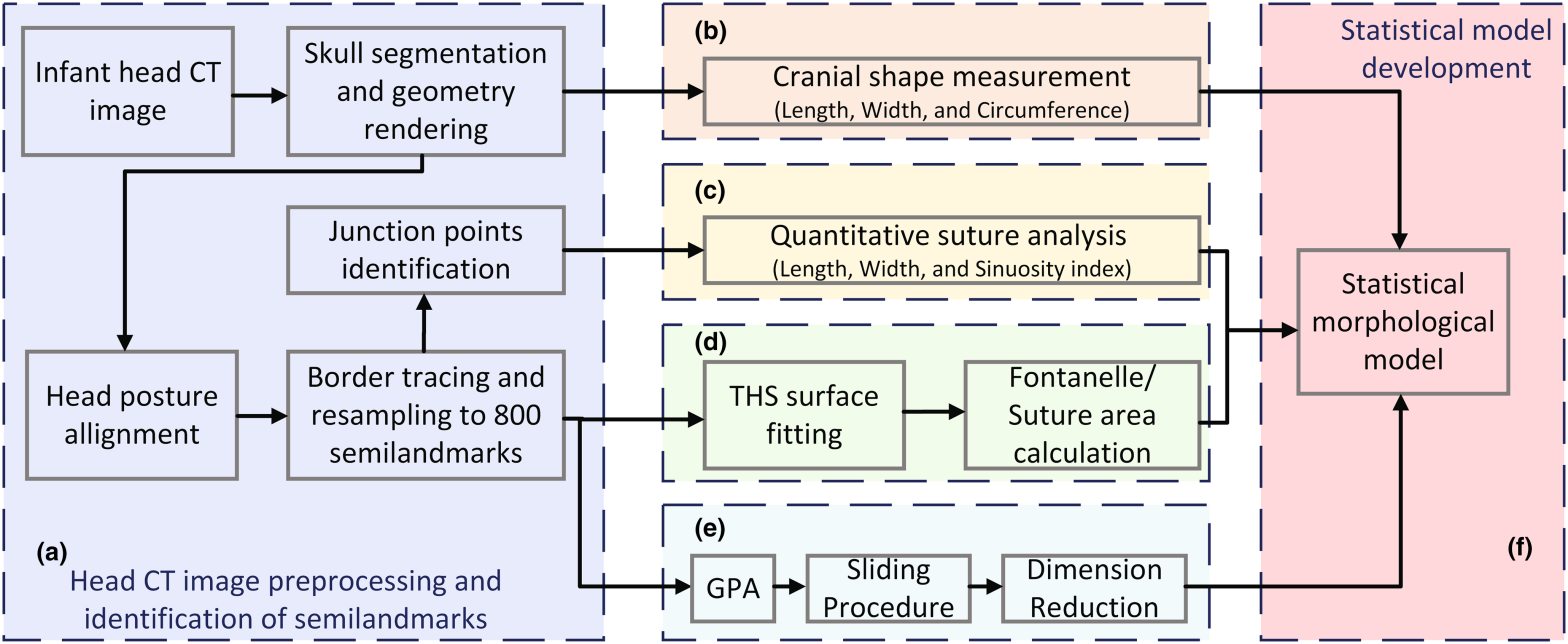
Framework for quantitative morphological analysis of infant cranial sutures and fontanelles, comprising primarily six parts: (a) preprocessing of infant head CT images, (b) cranial shape measurement, (c) measurement of cranial sutures, (d) surface reconstruction, (e) dimension reduction (GPA stands for Generalized Procrustes Analysis), and (f) development of a statistical model.

Six parts are included in the proposed framework of infant cranial sutures and fontanelles, as seen in Figure 1.

### 2.1 Head CT image preprocessing and identification of semilandmarks

High‐resolution full‐body CT scans from 72 infants aged 2–4 months were acquired from the New Mexico Decedent Image Database (Edgar et al., 2020), and only head part was used in this study. CT data of each subject was composed of a set of 512*512 images, and the slice thickness was 1 mm with 0.5 mm overlap, leading to a resolution range from 0.3*0.3*0.5 mm^3^ to 0.8*0.8*0.5 mm^3^. Among all CT scans, 69 subjects were selected with normal developmental cranial sutures and the absence of cranial fracture and dislocation. All CT images were processed in 3D slicer (Fedorov et al., 2012) with the following procedure.

**FIGURE 2:**
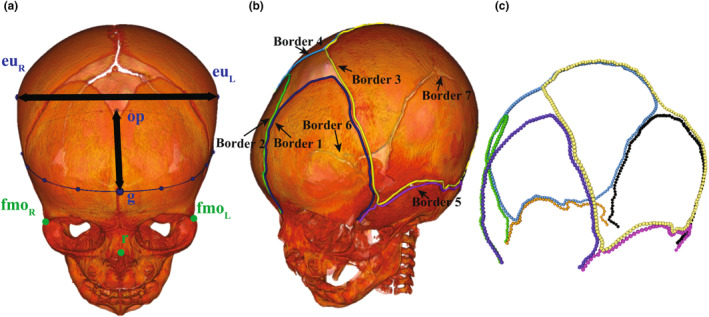
Head CT image preprocessing and semilandmarks identification illustrated with one subject. (a) Segmented skull with three anatomical points of rhinion (r), frontomalare orbitale right (${\mathrm{fmo}}_{R}$), and frontomalare orbitale left (${\mathrm{fmo}}_{L}$) for head posture alignment. The cranial shape was measured by locations of glabella (g), opisthocranion (op), and eurion (eu) on the cranial surfaces. (b) Border curves by manual tracing edges of sutures and fontanelles. Border 1, border 2, and border 7 were then resampled to 100 semilandmarks. Border 3, 4 were resampled to 200 semilandmarks, and border 5, 6 were resampled to 50 semilandmarks. (c) In total 800 semilandmarks were used to calculate morphological traits of sutures and fontanelles.

**FIGURE 3:**
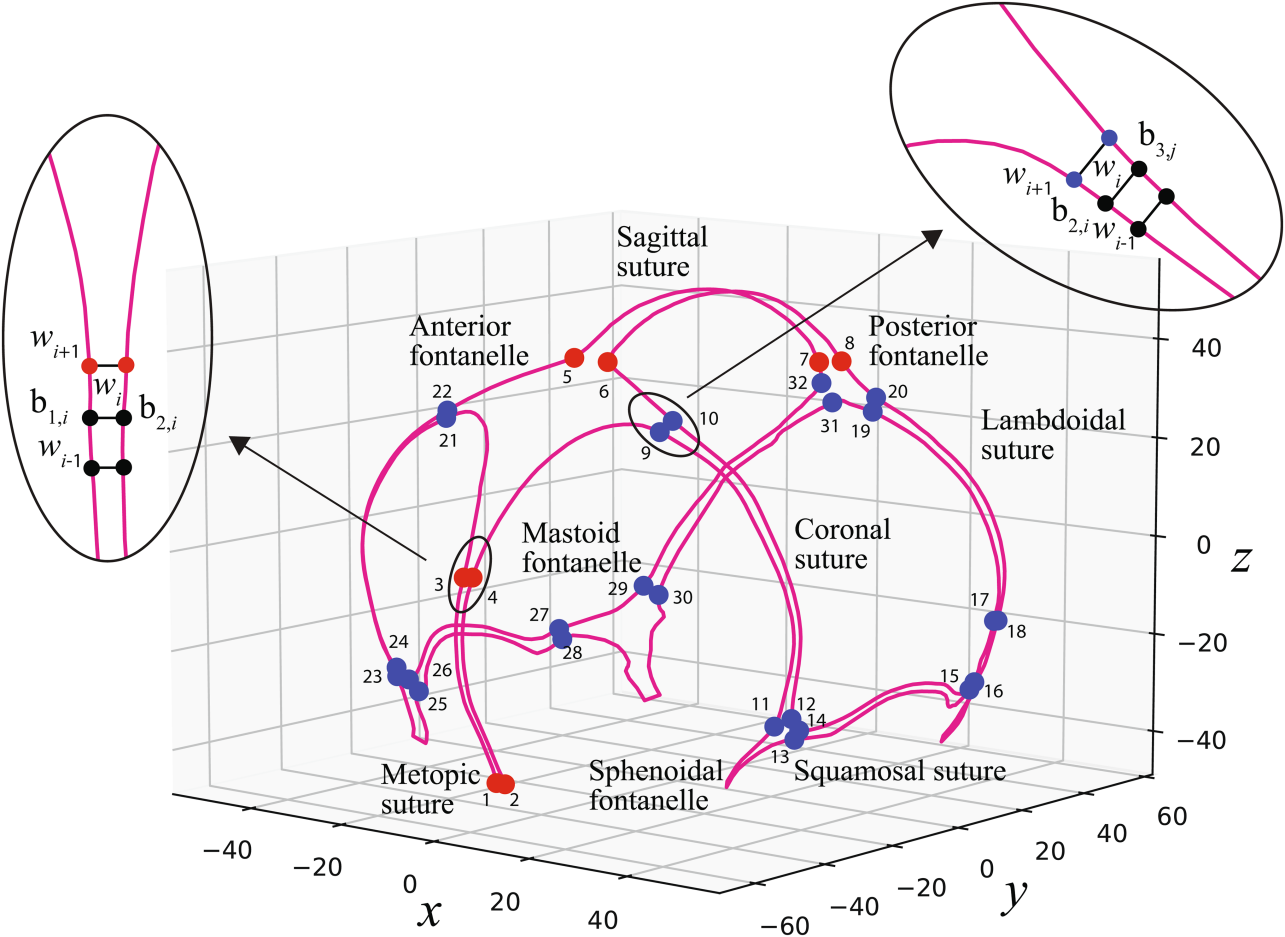
Nine parts of sutures and fontanelles including metopic suture, anterior fontanelle, coronal suture, squamosal suture, sagittal suture, lambdoidal suture, posterior fontanelle, sphenoidal fontanelle, and mastoid fontanelle were determined by 32 junction points. The metopic suture begins at the nasion, which is also the starting point of border 1 and border 2. The sagittal suture begins at the corner point of parietal bones, which is also the starting and ending point of border 3 and border 4. The ending points of both metopic suture and sagittal suture were identified based on the width variance of semilandmarks on their borders. The rest 24 junction points located at the lateral (in blue) were also identified by width variance, which determined the endpoints of the rest sutures. This figure uses the average morphology of suture and fontanelle generated by the mean PC scores of the 69 subjects.

1. Acquisition geometry regularization: The acquisition geometry regularization was activated before loading the CT image data to avoid the potential gantry tilt distortion.
2. Skull segmentation and geometry rendering: The CT scans were 3D‐rendered in bone window and segmented with a threshold of 275 Hounsfield Units (HU) to show only the infant cranial bones.
3. Head posture alignment: The head position and posture of each subject from the original CT images are different. To eliminate the positional difference, three anatomical points were manually annotated for all subjects rendered in bone window, and then all subjects were rigidly aligned to a reference sample using an integrated method by Fiducial Registration Wizard package (Ungi et al., 2016) based on the location of these three anatomical points, as shown in Figure 2(a).
4. Border tracing and resampling to 800 semilandmarks: The borders of the sutures and fontanelles were manually identified along the edges of the different segmented bony plate surfaces. For each subject, the border curve was then uniformly resampled to consist of an equal number of semilandmarks, evenly distributed between the two endpoints (Figure 2(b)). In total 800 semilandmarks were extracted to represent the geometry of sutures and fontanelles based on the segmented image, as shown in Figure 2(c).
5. Junction points identification: Nine parts of cranial sutures and fontanelles were determined based on 32 junction points (JPs), as shown in Figure 3. Since the border1 and border2 started from the nasion, the starting points of metopic suture (MS) were same with the starting point of border1 and border2 (JP1, JP2). To determine the junction points (JP3, JP4) between the MS and anterior fontanelle (AF), the width between the left and right frontal bones was calculated. Considering the AF exhibits a rhombus shape and experiences more pronounced width changes in comparison to the MS, the junction points adhere to the Equation 1. The following criterion on the left was applied to exclude semilandmarks exhibiting minimal width changes (less than *k* mm), the right equation ensured that there was a drastic change in width at the intersection of the MS and the AF. The searching process began at the starting point of the MS. Only the final set of points that satisfied the given equation were identified as the junction points between the suture and the fontanelle (*jp* = *jp* [−1], where the variable ‘*jp*’ stored the indices of all semilandmarks on border 1 that meet the criteria in python). Sagittal suture (SaS) began at the corner point of parietal bones in our framework (JP5, JP6). Similar to the endpoint of MS, the width between the left and right parietal bones was calculated, and the last set of points (JP7, JP8) that satisfied the following criteria was considered as the junction point between SaS and posterior fontanelle (PF). Because the width change at the junction of SaS and PF was greater than the width change between MS and AF, the value of ‘*k*’ in the equation was set to 0.18 for JP5 and JP6, and 0.15 for JP3 and JP4.

(1) $$w_{i+1}-w_{i}>k\&\left(w_{i+1}-w_{i}\right)>10^{*}k\left(w_{i}-w_{i-1}\right)$$

The blue junction points (JP9‐32) were identified according to the following criterion, where the value of *m* was set to 0.4.

(2) $$\left(w_{i+1}-w_{i}\right)-\left(w_{i}-w_{i-1}\right)>m$$

Owing to the scarcity of pertinent literature studies, the values of parameters *k* and *m* were selected through trial and error guided by the results of the conducted sensitivity analysis. The sensitivity analysis and discussion of different parameters were included in the supplementary materials.

### 2.2 Suture and fontanelle morphology measurement

The suture length was calculated as the average length of both sides. For metopic and sagittal suture, the suture widths were defined as the average width of all suture semilandmarks, and the MS width of the *i*th semilandmark was calculated as the distance between the *i*th semilandmark on border1 (${\mathrm{SL}}_{1,i}$) and the i th semilandmark on border2 (${\mathrm{SL}}_{2,i}$). For other sutures, due to the complex shape and asymmetry, the width was defined as the average shortest distance from a semilandmark on one border to another border. For instance, the coronal suture (CS) width of *i*th semilandmark on border 2 was defined as the distance between ${\mathrm{SL}}_{2,i}$ and ${\mathrm{SL}}_{3,j}$, where ${\mathrm{SL}}_{3,j}$ was the semilandmark which had the shortest distance to ${\mathrm{SL}}_{2,i}$ on border3 (Figure 3). The degree of suture interdigitation was quantified using the sinuosity index (SI), which indicated the deviation of the cranial suture from the straight line. Referring to the same measurement in literature, the suture SI was calculated by dividing the suture length by the suture distance from beginning to end (Adamski et al., 2015; Jaslow, 1990).

### 2.3 3D surface reconstruction for area calculation

**FIGURE 4:**
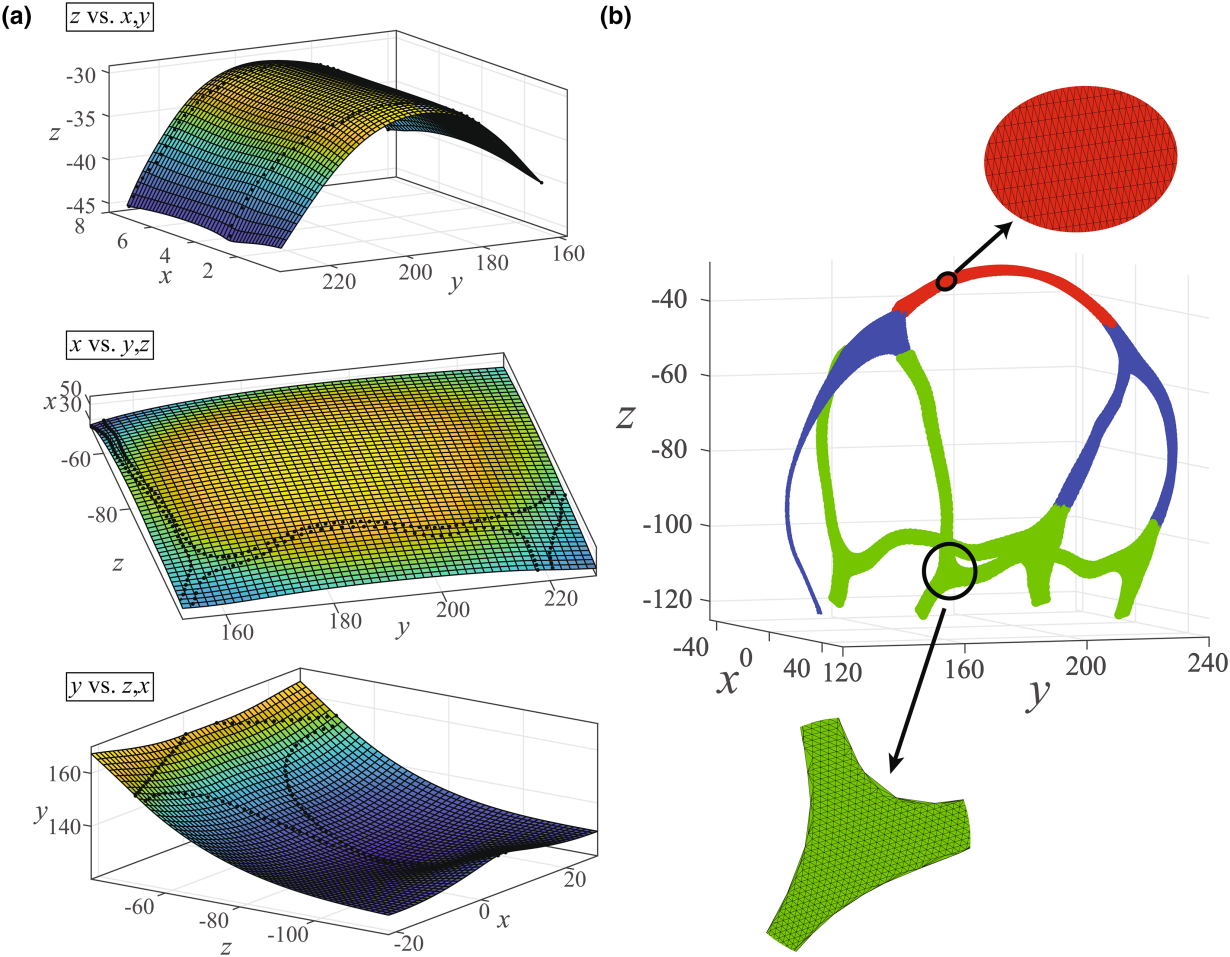
3D surface reconstruction of sutures and fontanelles. (a), Fitted surfaces of superior, lateral, and anterior–posterior portions based on the X‐Y, Y‐Z, Z‐X plane. (b), Surface reconstruction and triangulation. (The superior, anterior–posterior, and lateral portions are shown in red, blue, and green.)

Thin‐plate‐spline interpolant fitting method was used to reconstruct the surface of sutures and fontanelles based on the identified 800 semilandmarks. Because of the complex 3D shape of infant sutures and fontanelles, surfaces of superior, lateral, and anterior–posterior portions were individually fitted based on the X‐Y, Y‐Z, and Z‐X plane (Figure 4(a)). The five reconstructed 3D planes were subsequently represented as dense point clouds (Figure 4(b)), followed by triangulation into densely packed triangular elements to calculate the total surface area of sutures and fontanelles using Heron's formula (Equation 3)

(3) $$S=\sum_{i=0}^{m}\sqrt{s\left(s-a\right)\left(s-b\right)\left(s-c\right)}$$

where m is the number of triangular elements, a,b,c are the lengths of the sides of each triangle, $s=\left(a+b+c\right)/2$. The average side length of triangular elements was 0.3 mm for all subjects.

### 2.4 Cranial shape measurement

The cranial shape was represented by three parameters (length, width, and circumference). To facilitate accurate measurements, the original CT images were initially rotated in posterior–anterior and inferior–superior directions to make the coronal and sagittal planes consistent with the anatomical planes. Subsequently, the degree of left–right rotation was adjusted to make the glabella and the opisthocranion in the same axial plane of the 3D Slicer. Referring to (Allanson & Hennekam, 1997), the cranial length was measured from the anatomical point glabella (g) to the opisthocranion (op), and the cranial width was measured as the linear distance between the left and right erionu. The cranial circumference was defined as the occipitofrontal circumference (OFC), which was often used as an index in clinical practice to evaluate skull growth (Rijken et al., 2015). OFC was measured as a curve length through the g and op in the axial plane, as shown in Figure 2(a).

### 2.5 Dimension reduction

Generalized Procrustes Analysis (GPA) was first employed to achieve precise alignment of the semilandmarks for all subjects. In this study, GPA was conducted in the form space, utilizing all 800 semilandmarks to ensure the normalization of orientation and position across all subjects without changing the orginal size. The allocation of semilandmarks was initially set as equidistant points for the calculation of morphological traits, as mentioned in Section 2.1. However, this method is considered as straightforward but inherently subjective way to distribute semilandmarks, and can lead to significant stastistical inconsistencies and visualization artefacts (Gunz et al., 2005). To address these issues, the equidistant semilandmarks were subsequently slided slightly along border curves, ensuring geometric homology among the semilandmarks across all subjects. This sliding procedure was conducted using the R package *Morpho* (Schlager, 2017), and the sliding algorithm minimized the bending energy of a triplet of thin plate splines. This sliding procedure has been the standard approach in geometric morphometrics by now (Gunz et al., 2019; Liang et al., 2023; Perez et al., 2006).

The high dimensionality of raw data is generally redundant due to a large number of semilandmarks characterizing a sample. There are several dimensionality reduction methods, including the classic principal component analysis (PCA) method, kernel PCA, as well as deep‐learning‐based methods such as variational autoencoder (VAE). Next, we evaluated and compared all above mentioned methods for their performance in capturing the morphology of sutures and fontanelles.

#### 2.5.1 Classic principal component analysis

Principal component analysis (PCA), as one of the most popular methods of data dimensionality reduction, has been widely used in statistical shape models by projecting data from high‐dimensional space to low‐dimensional space through certain linear transformations without losing much information of the original data (Li et al., 2022; Mercan et al., 2020; Wei et al., 2022). To make the input data comparable and increase the model quality, semilandmark data was standardized by scaling three coordinate values to distribute around a mean of zero with a standard deviation of one before performing PCA and regression analysis. In this study, the number of subjects was *N* = 69, and each subject had 800 discrete points in 3D space, therefore the standardized geometry matrix *G* had a dimension of 69*2400. According to the PCA theory, *G* can be eigendecomposed as

(4) $$G=Z\left(G_{0}\right)=\mathrm{SP}$$

where $G_{0}$ is the original geometry matrix, *Z* is the standardization operator, *S* is the principal component score matrix with a dimension of 69*2400, and *P* is the eigenvalue matrix with a dimension of 2400*2400. For reducing the scale of dimension, the PCA for the first *k* principal components is in the form as

(5) $$G^{*}=S_{k}P_{k}$$

where $G^{*}$ is an approximation of G, $S_{k}$ is the first k columns of S with the dimension of 69*k, and $P_{k}$ is the reduced matrix composed of the first k eigenvectors of G with a dimension of k*2400. The geometry of the i‐th subject can be approximated by

(6) $$g_{0}^{*}=Z^{-1}\left(P_{k}^{T}S_{\mathrm{ik}}^{T}\right)$$

where $Z^{-1}$ is the inverse standardization operator, $P_{k}^{T}$ is the transpose of the $P_{k}$, and $S_{\mathrm{ik}}^{T}$ is the transpose of the i‐th row of $S_{k}$.

#### 2.5.2 Kernel PCA and deep learning based method

Kernel PCA (Schölkopf et al., 1997) uses a kernel function to project the dataset into a higher dimensional feature space to make it linearly separable. In addition to the classical PCA, five different kernel PCA (linear, cosine, rbf, sigmoid,and laplacian kernels) were evaluated in the present study.

Based on the neural network, variational autoencoder (VAE) can also be used to reduce the dimension of original data to latent space (Kingma & Welling, 2013). In the present study, two different VAEs (dense layers based and convolutional neural networks based [Eivazi et al., 2022]) were compared with the classical PCA. The theories of kernel PCA and VAE are briefly introduced in the Appendix A.

#### 2.5.3 Evaluation and comparison of reduction methods

All the dimension reduction methods were evaluated by the method of 10‐fold cross‐validation (Fushiki, 2011). The coordinates of sliding semilandmarks for the 69 subjects were randomly divided into 10 groups (9 groups with 7 subjects and 1 group with 6 subjects), and one of the groups was selected as the test group while the remaining 9 groups were used to train the reduced model as the training data. The generalization ability of different models was evaluated by the average location mean deviation (LMD) error of the training model on the test data,

(7) $$\mathrm{LMD}\left(A,B\right)=\frac{1}{n}\sum_{i=0}^{n-1}\left|i\left[A\right]-i\left[B\right]\right|$$

where *A*,*B* are the original data and the predicted data, *i* is the sequence value, and *n* is data dimension.

### 2.6 Regression for statistical model and evaluation

The classic PCA was employed in this section due to its demonstrated superior performance compared to the other evaluated methods (details can be found in Section.3.1). PCA projects the data onto a low‐dimensional orthogonal space, which is amenable to regression analysis. In this section, a linear regression model was used to develop the relationship between PC scores and cranial shape parameters (width, length, circumference) and suture morphological parameters (SI, surface area) (Mercan et al., 2020),

(8) $$S_{k}=\mathrm{CF}$$

where F is a feature matrix, and the coefficient matrix C can be estimated using standard least‐squares techniques by taking the Moore‐Penrose pseudoinverse $F^{+}$ of F such that

(9) $$C=S_{k}F^{+}$$

Therefore, a statistical‐based geometry of the i‐th subject can be generated by

(10) $$g_{0}^{*}=Z^{-1}\left({P_{k}^{T}}^{*}{\left(\mathrm{CF}\right)}_{\mathrm{ik}}^{T}\right)$$

The quality of different reduction methods was evaluated by 10‐fold cross‐validation in the previous section. To further evaluate the reliability and applicability of the framework for predicting the infant cranial suture, the quality of the statistical models was comprehensively evaluated using leave‐one‐out cross‐validation (LOOCV), which involved visually and quantitatively comparing the predicted suture shape with the real suture shape. The actual cranial size and suture parameters of each subject were used as input for the statistical model generated by the remaining subjects. Subsequently, the average predicted length, width, and SI of different suture parts were compared with the real CT scans measurement, and the LMD errors of different sutures and fontanelles between the predicted suture morphology and the real suture morphology were calculated.

## 3 RESULTS

### 3.1 Performance of different dimension reduction methods

With the same reduced dimension, the average LMD errors of the different dimension reduction methods mentioned in Section 2.5 on training data and test data were reported in Table 1. Overall, the performance of the classic PCA and linear kernel PCA was similar. However, the LMD error on the test data for all non‐linear dimension reduction methods was larger than the error observed with linear methods, which indicated that nonlinear methods generally exhibit inferior generalization ability for this statistical morphology model. Among these nonlinear methods, the convolutional neural networks based VAE model had the minimum LMD error on the training data, which suggested the most overfitting during the training phase. Since this study focused more on the generalization ability of the statistical regression model, the classic PCA, which had a comparable error of the linear kernel PCA but had the most simplicity and interpretability, was finally chosen.

**TABLE 1:** Average LMD error of different dimension reduction methods on the training data and the test data.

	Average LMD error on training data (mm)	Average LMD error on test data (mm)
Classic PCA	0.4352	0.8907
Linear Kernel PCA	0.4437	0.8805
Cosine Kernel PCA	0.9077	1.2422
Rbf Kernel PCA	1.9309	2.0545
Sigmoid Kernel PCA	2.0757	2.1169
Laplacian Kernel PCA	1.765	2.0126
Convolution Layer VAE	0.2144	1.5129
Dense Layer VAE	1.655	1.967

### 3.2 Morphological model of infant suture and fontanelle

**FIGURE 5:**
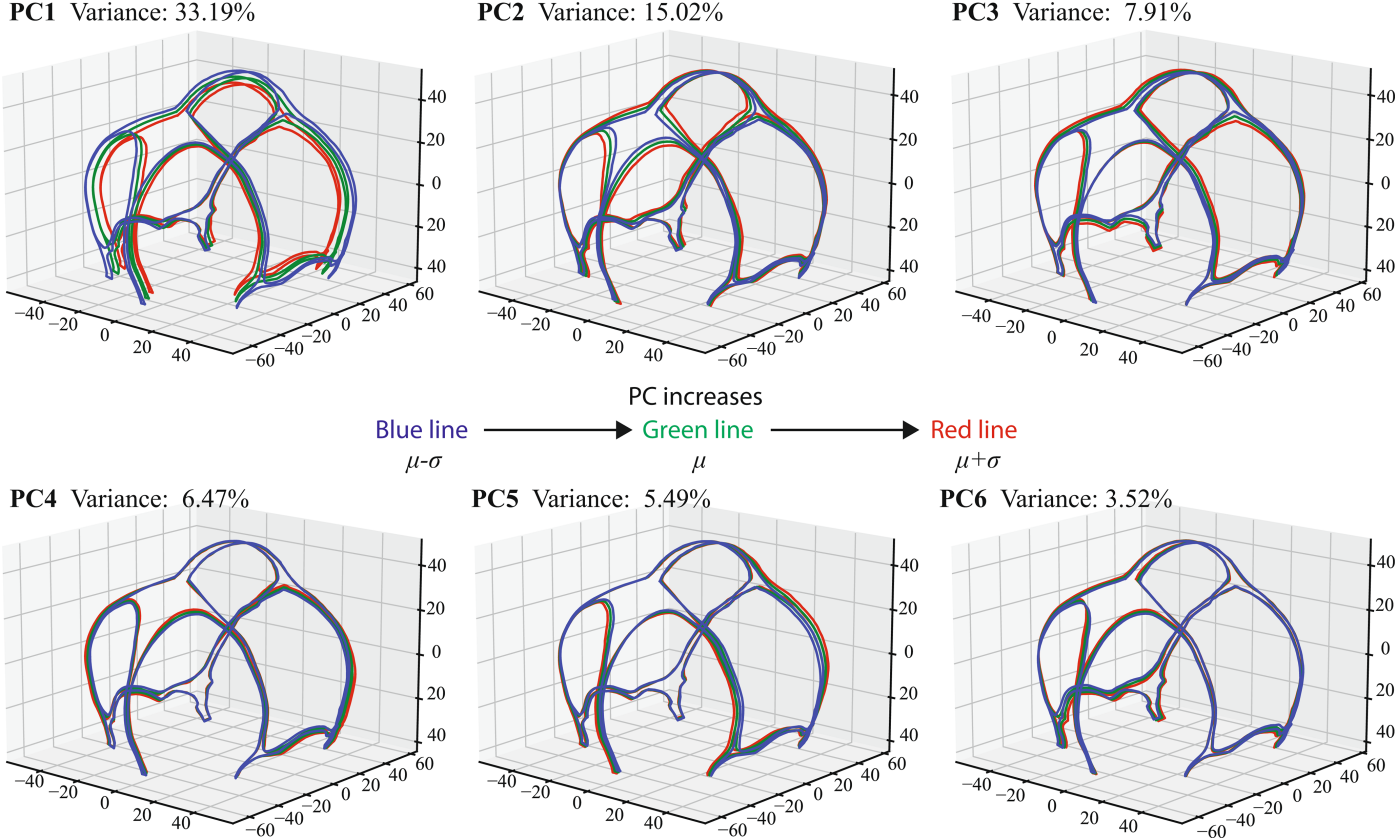
Visualization of infant cranial suture shape variation captured by the first six principal components using the corresponding PC scores in standard deviations around the mean.

**FIGURE 6:**
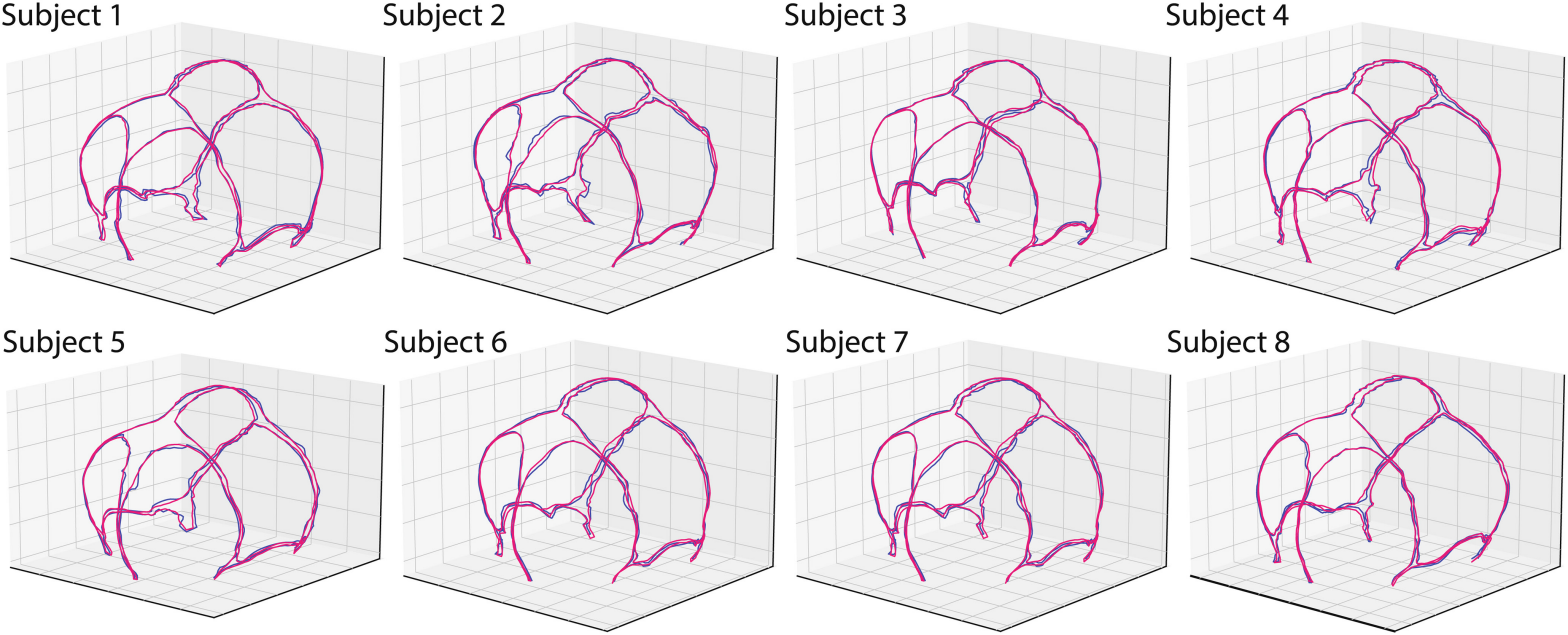
The comparison between the reconstructed suture morphology using the first 30 PCs (pink) and the real suture geometry (blue) of the first eight subjects.

The suture morphologies and the corresponding 800 equivalent semilandmarks for all subjects were identified. In our model, the reduced number of principal components k was set as 30 because the first 30 principal components could account for 95% of the geometric variation. Among the 30 PCs, the first six PCs explained 33.19%, 15.02%, 7.91%, 6.47%, 5.49%, and 3.52% of the variation for sutures and fontanelles of 2–4 months infant, respectively. As visualized in Figure 5, PC1 mainly varied the overall size of suture morphology combined with the cranial size while the rest of the PCs varied the cranial sutures and fontanelles morphology without changing the cranial size significantly. The comparison between the reconstructed suture morphology with the first 30 PCs and the actual suture geometry from the first 10 CT images is displayed in Figure 6. The results showed good accuracy with an average LMD error of 0.4633 mm with 30 PCs describing the locations of 800 semilandmarks.

### 3.3 Cranial size and suture shape variation

**FIGURE 7:**
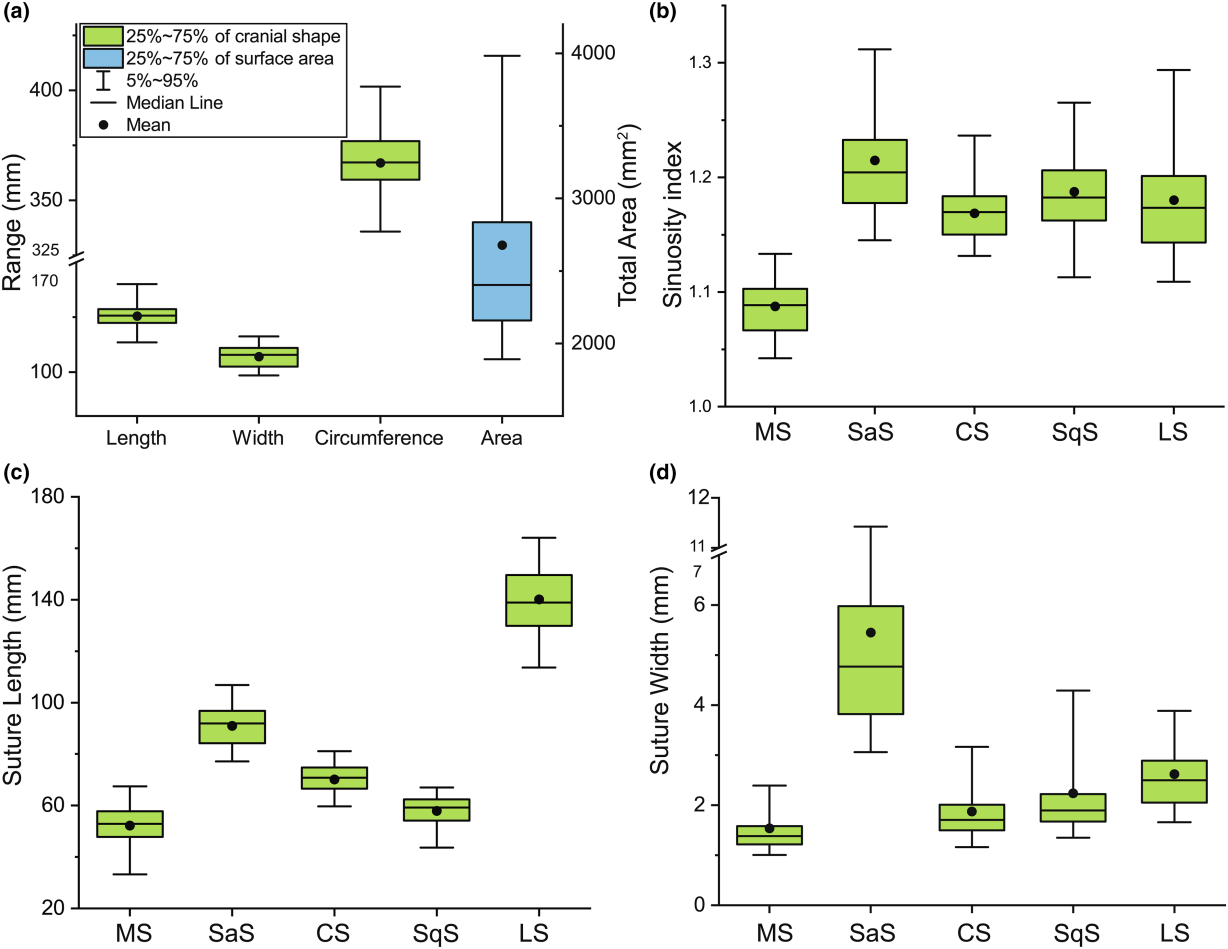
Box‐plot of the measured cranial and suture size for infants in 5th, 25th, 50th, 75th, and 95th percentiles, and average at 2–4 months. (a), The cranial shape parameters of length, width, and circumference combined with the total surface area of sutures and fontanelles. (b), Sinuosity index of various cranial sutures. (c), Length of various cranial sutures. (d), Width of various cranial sutures.

Figure 7 illustrates the box plots of measured cranial shape parameters (length, width, circumference) combined with cranial suture shape parameters (SI, length, and width) of subjects in this study. The average cranial length, width, and circumference of subjects were 125.44±7.61 mm, 107.03±6.90 mm, and 367.05±21.33 mm, respectively. The average cranial suture widths for metopic, sagittal, coronal, squamosal, and lambdoid sutures were 1.54±0.84 mm, 5.45±3.25 mm, 1.88±0.77 mm, 2.24±1.38 mm, and 2.62±0.94 mm. The average cranial suture lengths for metopic, sagittal, coronal, squamosal, and lambdoid sutures were 52.23±9.50 mm, 90.97±9.45 mm, 69.87±8.77 mm, 57.96±6.65 mm, and 140.06±15.33 mm. Among the various measured sutures in 2‐ and 3‐month‐old infants, the MS had been found to have the lowest average SI, shortest average length, and narrowest average width. On the other hand, the lambdoid suture was characterized by having the greatest length, and the SaS was distinguished by having the largest width and the most tortuous pattern.

### 3.4 Statistical model of infant suture and fontanelles aged 2–4 months

In the regression model of Equation 2.10, a relationship between the principal scores and the infant cranial shape and suture parameters was developed. The $R^{2}$ values of statistical infant suture models generated with the selected parameters are reported in Table.2.

**TABLE 2:** $R^{2}$ values of the PCAR models with cranial shape parameters, total surface area, suture sinuosity index, and their combination.

	L	W	C	S	SI	ALL
PC1	0.7419***	0.7698***	0.9562***	0.0685	0.0015	0.9682***
PC2	0.0208	0.0209	0.0013	0.377***	0.1723**	0.6097***
PC3	0.0004	0.0003	0.0006	0.4480***	0.1850**	0.6720***
PC4	0.07	0.0307	0.0031	0.0564	0.0028	0.4228***

*Note*: Significance (*p*‐value): <0.0001***, <0.001**.

Abbreviations: C, circumference; L, length; S, surface area; SI, average sinuosity index; W, width.

The PC scores, which could uniquely determine the morphology of infant suture and fontanelle, were influenced by multiple factors, such as cranial shape parameters, total surface area, and the SI of sutures. Corresponding to Figure 5, PC1 was highly correlated with cranial size (*p* < 0.0001) while PC2 (*p* < 0.0001) and PC3 (*p* < 0.0001) were significantly associated with a total surface area of sutures and fontanelles, and the average SI of cranial sutures primarily influenced the PC2 (*p* < 0.001) and PC3 (*p* < 0.001).

**FIGURE 8:**
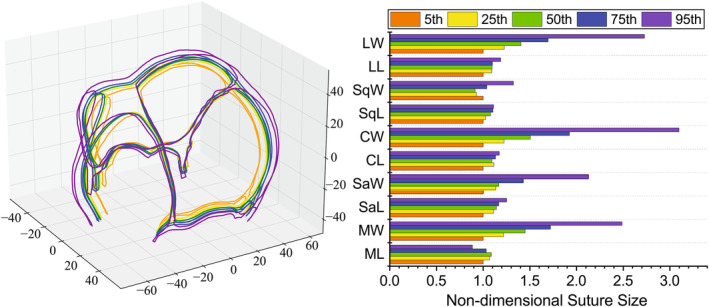
Suture morphology at the 5th, 25th, 50th, 75th, and 95th percentiles for infants aged 2–4 months. (a), Visualization (color in order is orange, yellow, green, blue, purple). (b), The quantitative variation of various non‐dimensional suture sizes (ML/W: Metopic suture length/width, SaL/W: Sagittal suture length/width, CL/W: Coronal suture length/width, SqL/W: Squamosal suture length/width, LL/W: Lambdoidal suture length/width).

The final statistical model employed a combination of all cranial shape parameters, area, and SI due to the highest $R^{2}$ value and the lowest p‐value from Table 2. Based on the developed regression model, the statistical suture morphologies of 5th, 25th, 50th, 75th, and 95th percentile infants were generated to reflect the variation of 2–4 months infant sutures (Figure 8(a)). The results indicated those with larger head circumference tend to have a larger fontanelle for infants aged 2–4 months. Figure 8(b) presents the results of variations in the length and width of the major sutures. To facilitate comparison among the different parameters, the data were normalized by dividing the corresponding value of the 5th percentile.

## 4 DISCUSSION

### 4.1 Developed quantitative morphological analysis framework

In this study, we reported a quantitative morphological analysis framework of infant sutures and fontanelles, which allowed for precise measurement of various parameters related to the suture and fontanelle morphology, including size, area, and sinuosity index. Additionally, we have developed a standardized method for distinguishing the boundaries between sutures and fontanelles based on variance in width changes.

Accurate identification and measurement of sutures and fontanelles can help researchers and pediatricians to better understand the mechanisms of cranial growth. In the past, the width of sutures was measured by manually determining several measurement points and calculating the average width of these points (Erasmie & Ringertz, 1976; Mitchell et al., 2011). The measurement of suture length was performed by calculating the straight‐line distance between the two endpoints of the sutures on a two‐dimensional plane (Sim et al., 2012). For the AF, Davies et al. used the index finger to determine four apices of the AF and evaluated the surface area by measuring the maximum anteroposterior and mediolateral linear lengths (Davies et al., 1975). However, these conventional 2D image‐based methods failed to account for the curvature of the sutures and fontanelles in the measurement, nor could they repeatably distinguish the boundary within a standard routine. The framework presented in this study aims to develop an automatic method for identifying distinct sutures and fontanelles based on the three‐dimensional characteristics of sutures, providing a more objective and reproducible measurement than previous methods. The developed framework will contribute to furthering research in the development and closure of cranial sutures while also providing a quantitative three‐dimensional representation of suture shapes to aid in the diagnosis and treatment of craniosynostosis.

### 4.2 Infant suture morphology

The past studies on pediatric head morphology primarily focused on head contour and skull shape, with limited research focusing on the morphology of infant sutures. In a previous study by Li et al. (Li et al., 2015), the width and closure of sagittal sutures for 0–3 years of infants were investigated. In that statistic model, age was considered as the only predictor for sagittal suture morphology instead of the variation in the same age. Idriz et al. (Idriz et al., 2015) studied the closure time and the normal anatomy of pediatric sutures. To better represent and understand the morphological variations for infant sutures, an integrated suture morphology analysis framework including a statistical model and a quantitative suture measurement was developed in the present study. This framework enabled precise measurements of various parts of sutures and fontanelles, providing a valuable tool for developing more sophisticated statistical models. The results of suture morphological changes in different percentiles of infants aged 2–4 months suggested that there was a general positive correlation between cranial size and cranial suture width in infants of 2–4 months old while the 50th percentile had the lowest width of the SqS. Compared with suture width, the variability of cranial suture length among different percentiles was relatively low. Sagittal, cronoal, and lambdoidal sutures increased steadily from the 5th percentile to the 95th percentile while the MS length of the 50th percentile was the highest, which indicates there was no significant correlation between the length of the MS and overall head size. The current framework has enabled the revelation of novel morphological rules pertaining to cranial sutures, which have not been reported in previous studies.

The developed statistical morphology model for the 2–4 month infant can be used in a wide range of applications, notably in the field of computational biomechanics. Leveraging the detailed shape of sutures and fontanelles generated by the morphology model, statistical‐based FE models can be efficiently adapted from existing FE infant models to focus on the biomechanical impact of suture variations (Li, 2021; Li et al., 2021). Furthermore, the availability of quantitative morphology data for various major sutures enables a systematic evaluation of the influence of individual sutures on infant biomechanics. By predicting potential head injuries, these models may also contribute to forensic analyses concerning the compatibility of skull fractures with documented trauma. Undertaking individual assessments based on detailed anatomical information, including sutures and fontanelle size, offers a more robust approach compared to conclusions drawn solely from clinical experience at the group level.

### 4.3 Evaluation of the statistical regression model

**FIGURE 9:**
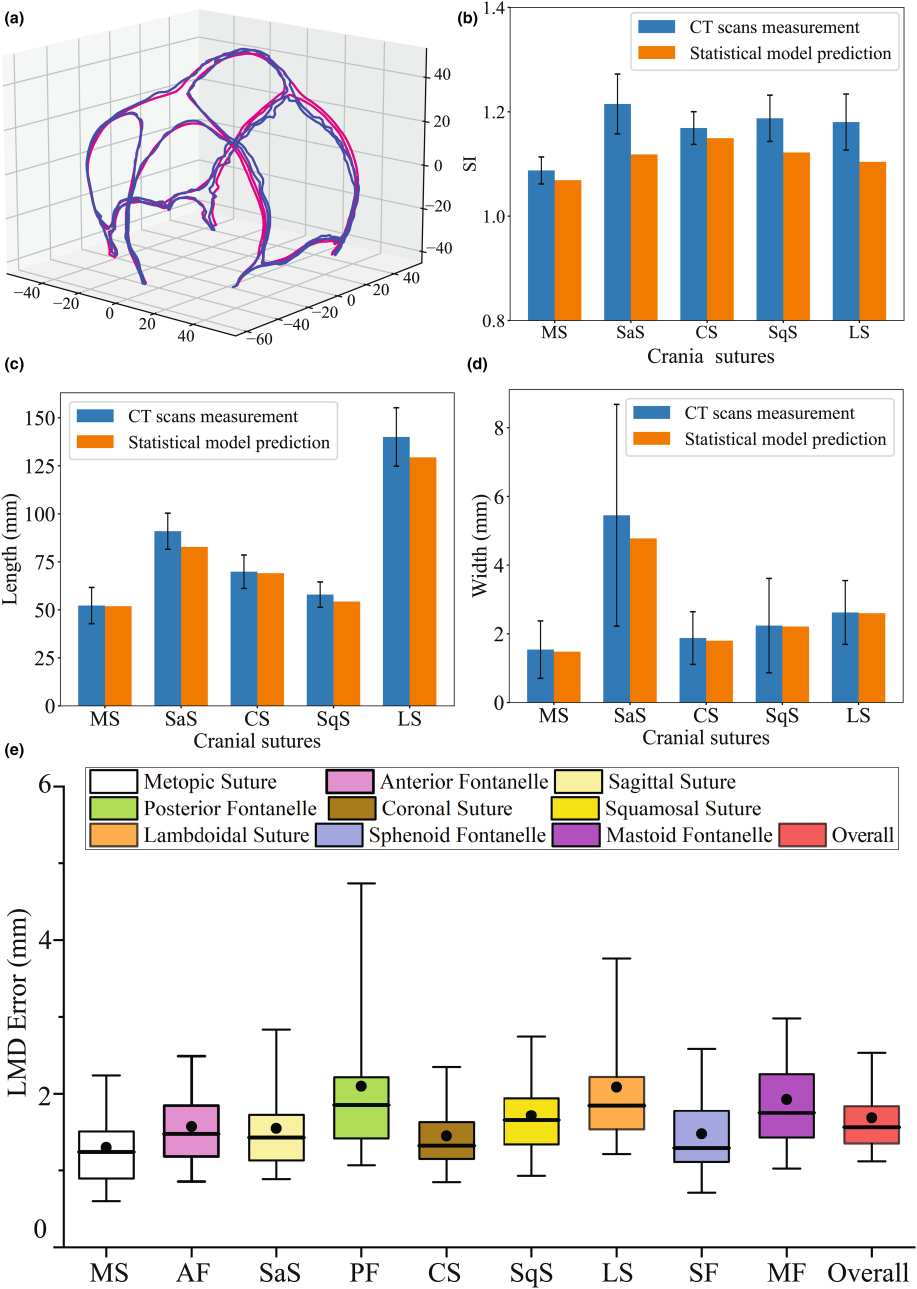
(a) Visual comparison between the predicted morphology (pink) and the real morphology from the CT image (blue). (b) The quantitative comparison of suture sinuosity index of 2–4 months old infants directly from the real CT images and predicted by the statistical model. (c) The quantitative comparison of suture length of 2–4 months old infants directly from the real CT images and predicted by the statistical model. (d) The quantitative comparison of suture width of 2–4 months old infants directly from the real CT images and predicted by the statistical model. (e) The box plot shows the LMD error for nine parts of sutures and fontanelles. Each box represents the median, mean, 5th, 25th, 75th, and 95th percentiles values.

By using LOOCV, Figure 9(a) shows a visual comparison of two subjects between the predicted morphology from the statistical regression model and the real morphology from the CT image. The predicted morphological characteristics of all subjects were estimated using input cranial shape and suture morphological parameters.

To further quantify the performance on different sutures and fontanelles, we calculated the LMD error of nine parts of all subjects, as shown in Figure 9(b). The average value of the overall LMD error was 1.6798 mm, the MS had the lowest LMD error with average value of 1.2969 mm while the PF had the highest average LMD error of 2.0974 mm. Besides that, the average cranial suture length and width predicted by the regression model were compared with the measured range of the real suture length and width from the original CT scans (in standard deviations around the average), as shown in Figure 9(c). The results demonstrated that the suture generated by the statistical model exhibited greater symmetry and less interdigitation compared to the actual suture, resulting in a slightly smaller predicted average suture size. Given that the test data can be considered unknown to our model, the generated suture morphology closely corresponded to the real suture for those subjects. The results showed that the generated suture morphology by the established regression model corresponded with the real suture for the 69 subjects, demonstrating the accuracy of the proposed framework.

### 4.4 Limitations

The suture analysis framework reported in this paper should be considered in light of certain limitations. First, the collection of 72 CT images for infants aged 2–4 months was limited due to the rarity of available infant CT head images, which may affect the quality of the statistical model. However, this framework is ready to be applied to CT images of infants across all ages when such data are available to systematically investigate the suture growth with age as the framework allows accounting suture closure by zero‐width. Second, in this study, the biological sex of subjects was not equally represented. Consequently, this study did not investigate the impact of gender on suture morphology. Finally, this suture morphology framework focuses solely on the major cranial sutures and does not consider the minor cranial sutures, due to their lack of connection with infant FE head models.

## 5 CONCLUSION

This study has introduced a quantitative morphological framework for the comprehensive analysis of infant suture and fontanelle. By utilizing 69 CT images of infants aged 2–4 months, the framework successfully identifies and quantifies variations in infant suture morphology. Our research confirms that PCA is the optimal dimension reduction method compared to other evaluated dimensional reduction methods. We have found that cranial size is significantly associated with the first PC variable, which account for 33% of the variance in infant suture and fontanelles while the surface area and sinuosity index are highly related to PC2, PC3 and PC4 (with a total explained variance of 29%). Furthermore, the verified developed statistical model for 2–4 month‐old sutures demonstrates the reliability and applicability of the framework, contributing to a better understanding of the variance in infant suture morphology.

In summary, this research provides valuable insights into comprehensive infant suture morphology and lays the foundation for future studies in this field. The collected data related to infant suture and fontanelles can serve as a reference for pediatricians and other researchers in this field. Additionally, this quantitative morphological analysis framework of infant cranial sutures and fontanelles can be readily implemented across different age groups of infants to investigate the growth rules of infant sutures.

## AUTHOR CONTRIBUTIONS

**Siyuan Chen**: Conceptualization, Methodology, Writing – original draft, Data processing, Coding, Validation.

**Svein Kleiven**: Conceptualization, Methodology, Writing – review & editing, Supervision.

**Ingemar Thiblin**: Conceptualization, Methodology, Writing – review & editing.

**Xiaogai Li**: Data collection, Conceptualization, Methodology, Writing – review & editing, Supervision.

## CONFLICT OF INTEREST STATEMENT

The authors declare no conflict of interest.

## Supporting information


Data S1:

Data S2:

## Figures and Tables

**FIGURE A1:**
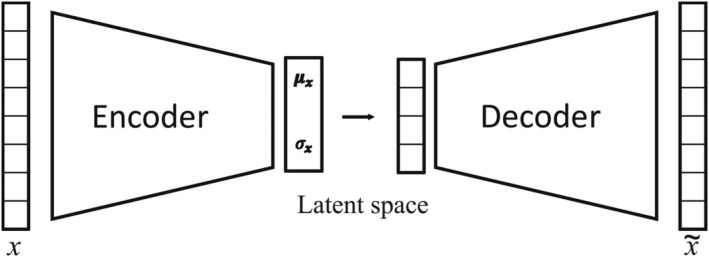
The structure of variational autoencoder.

## Data Availability

Source code of the developed framework has been integrated into an open‐source package named Infant Suture & Fontanelle Morphology Analysis Framework (ISFMA). The source code of ISFMA is freely available at https://github.com/siyuankth/ISFMA. The p value and $R^{2}$ value of the correlation between the first 30 PC scores of 69 subjects and a total of 31 morphological traits, including 3 cranial shape parameters, 13 area parameters, and 15 suture shape parameters, were provided in the supplementary materials.

## APPENDIX A

### A.1 Kernel PCA

Kernel PCA is a nonlinear extension of PCA which is based on the theory of Reproducing Kernel Hilbert Spaces (RKHS) (Berlinet & Thomas‐Agnan, 2011), where the original data X with a dimension of K×N is mapped to the high‐dimensional space Ϝ with a dimension of D×N using a nonlinear projection ϕ.

The variance covariance matrix $C_{Ϝ}$ in Ϝ has a dimension of D×D, which is

(A.1)
$$C_{Ϝ}=\frac{1}{N}\phi \left(X\right){\left[\phi \left(X\right)\right]}^{T}=\frac{1}{N}\sum_{i=1}^{N}\phi \left(x_{i}\right){\left[\phi \left(x_{i}\right)\right]}^{T}$$

The eigenvector p of $C_{Ϝ}$ can be represented as a linear combination of $\phi \left(x_{i}\right)$

(A.2)
$$p=\sum_{i=1}^{N}\alpha_{i}\phi \left(x_{i}\right)=\phi \left(X\right)\alpha$$

where $\alpha ={\left[\alpha_{1},\alpha_{2},\ldots ,\alpha_{N}\right]}^{T}$. Combined the Equation A.2 with $C_{Ϝ}p=\lambda p$, where λ is the eigenvalue of $C_{Ϝ}$, there is

(A.3)
$$\phi \left(X\right){\left[\phi \left(X\right)\right]}^{T}\phi \left(X\right)\alpha =\mathrm{N\lambda \phi}\left(X\right)\alpha$$

Multiply both sides by the matrix ${\left[\phi \left(X\right)\right]}^{T}$,

(A.4)
$${\left[\phi \left(X\right)\right]}^{T}\phi \left(X\right){\left[\phi \left(X\right)\right]}^{T}\phi \left(X\right)\alpha =\mathrm{N\lambda}{\left[\phi \left(X\right)\right]}^{T}\phi \left(X\right)\alpha$$

Define $K={\left[\phi \left(X\right)\right]}^{T}\phi \left(X\right)$ with the dimension of N×N, where $K_{\mathrm{ij}}=\phi {\left(x_{i}\right)}^{T}\phi \left(x_{j}\right)$. Equation A.4 can be written as

(A.5)
K⋅Kα=NλKα

Then, the eigenvalue λ can be solved through

(A.6)
Kα=Nλα

The kernel functions used in this paper are listed in Table A1.

**TABLE A1:** Five different kernel used in this study $\left(\gamma =0.05,c_{0}=1\right)$.

Linear kernel	$k\left(x,y\right)=x^{T}y$
Cosine similarity kernel	$k\left(x,y\right)=\frac{{\mathrm{xy}}^{T}}{∥x∥∥y∥}$
RBF kernel	$k\left(x,y\right)=\exp\left(-\gamma ∥x-y{∥}^{2}\right)$
Degree‐2 polynomial kernel	$k\left(x,y\right)={\left(\gamma x^{T}y+c_{0}\right)}^{2}$
Degree‐3 polynomial kernel	$k\left(x,y\right)={\left(\gamma x^{T}y+c_{0}\right)}^{3}$

### A.2 VAE

VAE is a type of unsupervised deep learning model that consists of the parts of encoding and decoding, as shown in Figure A1. In a VAE model, the original data will be encoded into a latent space with a lower dimension. Subsequently, those points will be mapped back to the original dimension of space. The model updates the parameter values with each epoch to make the reconstructed data similar to the original data. The loss function of VAE consists of reconstruction loss and regularization loss,

(A.7)
$$\text{loss}=∥x-\tilde{x}{∥}^{2}+\mathrm{KL}\left[N\left(\mu_{x},\sigma_{x}\right),N\left(0,1\right)\right]$$

where the former term $∥x-\tilde{x}{∥}^{2}$ is the reconstruction loss, KL is the loss of Kullback–Leibler divergence term, $N\left(\mu_{x},\sigma_{x}\right)$ is the latent distribution, and $N\left(0,1\right)$ is the standard Gaussian distribution.
