# Supplementary material for: Quantitative morphological analysis framework of infant cranial sutures and fontanelles based on CT images
Source: J Anat. 2024 May 9;245(3):377–91. doi: 10.1111/joa.14056 (PMC11306764; doi:10.1111/joa.14056)
Supplement: Supplementary file 1 — Data S1: [file JOA-245-377-s002.docx]

Sensitivity analysis of the selected parameters

**The junction point determination**

We did some sensitivity analysis before we chose the value of *k* and *m*. While defining the junction of sutures and fontanelle, we conducted an extensive literature search. Unfortunately, we found that none of the references provided a clear delineation of the endpoints of the metopic suture. However, for our computational purposes, it is crucial to distinguish these junctions, especially when measuring the length of different sutures.

To address this challenge, we incorporated mathematical formulas to regulate the identification of these junctions. We acknowledged that the formulation and parameters we used lack a theoretical basis. We recognized that there may be alternative mathematical formulas that could also effectively distinguish these junctions. We employed variations in width as a criterion for selecting junction points because we observed significant differences in width between the suture and the fontanelle. And our choice of formula was based on its successful application to all of our subjects, using the approach of trial-and-error. Throughout our research, we experimented with various parameters, but it was noted that the final selection of parameters must apply to all subjects to distinguish suitable junction point of sutures and fontanelles rather than majority of subjects. Below figures showed the junction points determined with other parameters (points shown in red while junction points under parameters used in our study were shown in black for comparison).

***k* of 0.15 for JP3 and JP4**

If *k* was set to 0.14, for some subjects, the identified JP3 and JP4 (shown in red) would be slightly higher in the direction towards the calvaria compared with the locations of JP3 and JP4 under the *k* of 0.15 (shown in black). In our assessment, we found that this particular segmentation was relatively unreasonable due to the significant variation in width near the junction of the metopic suture and the anterior fontanelle. Consequently, this resulted in calculated metopic suture dimensions that exhibited both increased length and greater width. *k* values less than 0.14 yielded similar results.


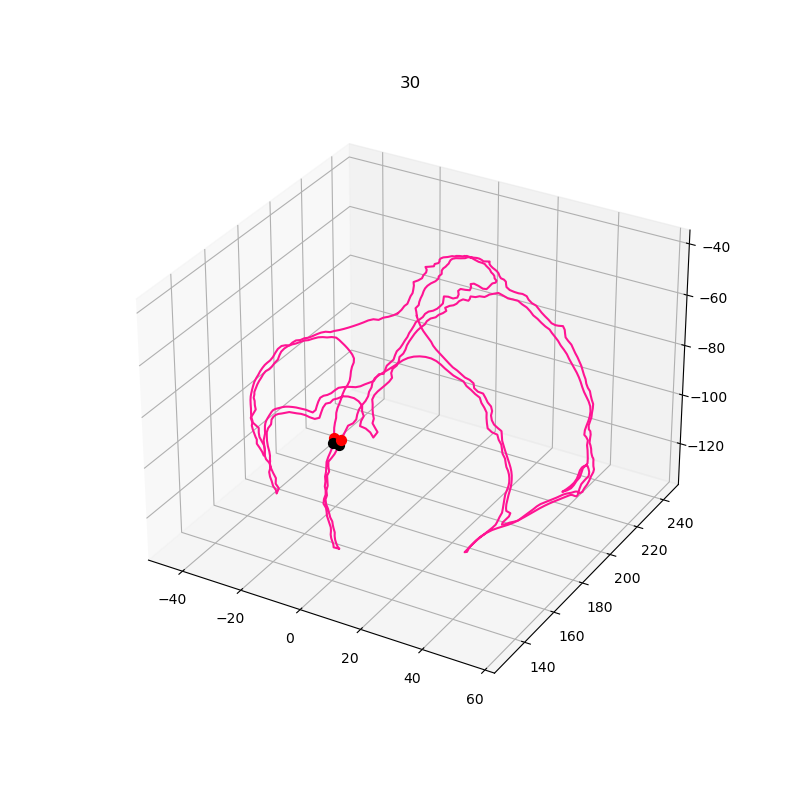

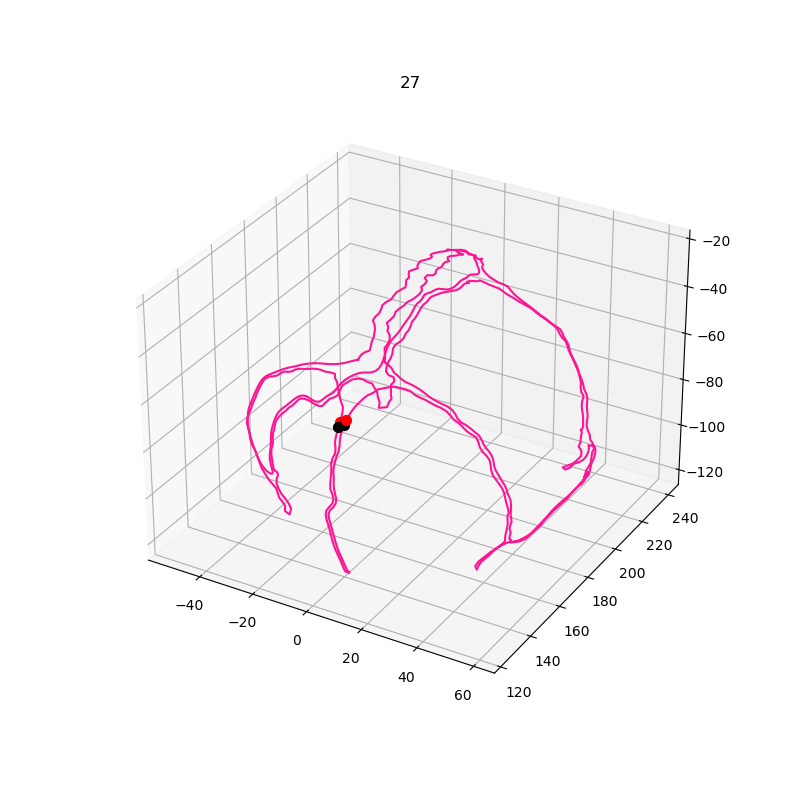

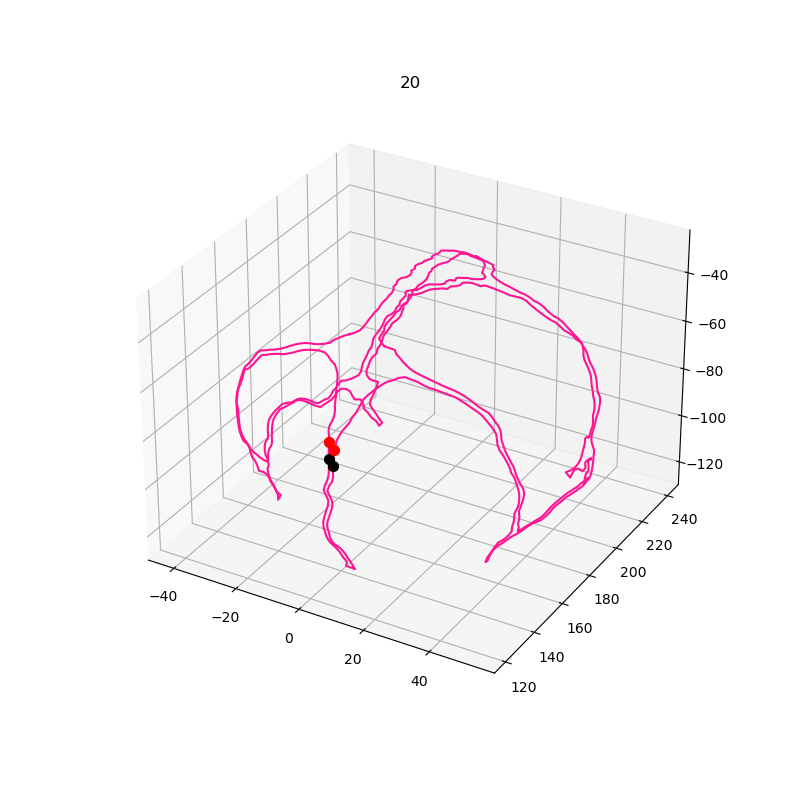

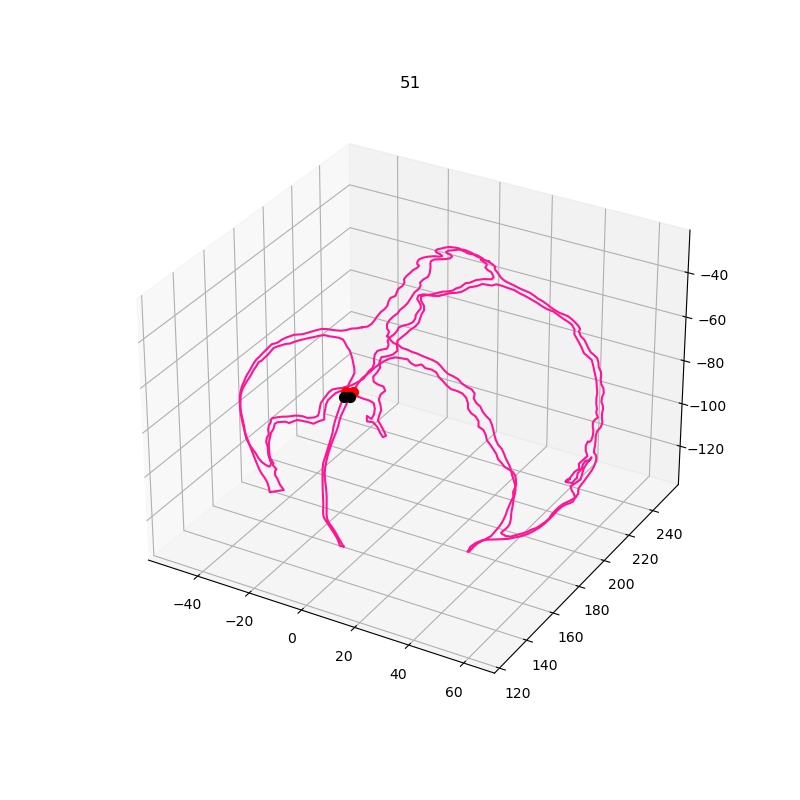


**Figure 1** The comparison of JP3 and JP4 under different *k* values (points of *k* =0.14 were shown in red, and point of *k*=0.15 were shown in black).

In contrast to smaller values of *k*, larger *k* values resulted in the identification of JP3 and JP4 at lower position, leading to a narrower width at the junction of the anterior fontanelle. Consequently, this reduced the measured length and width of metopic sutures. *k* values more than 0.16 of metopic suture yielded similar results.


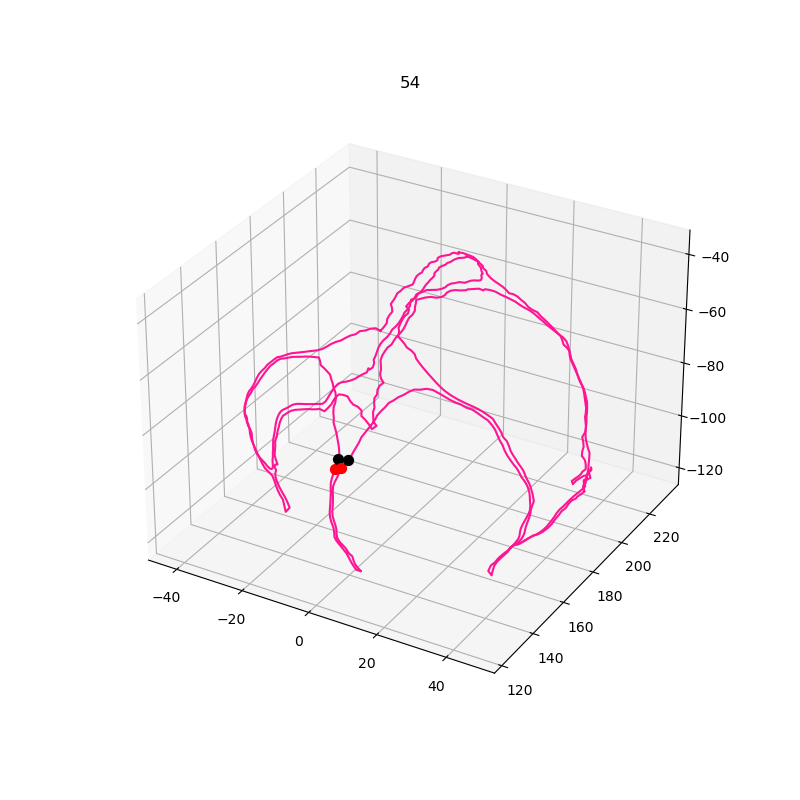

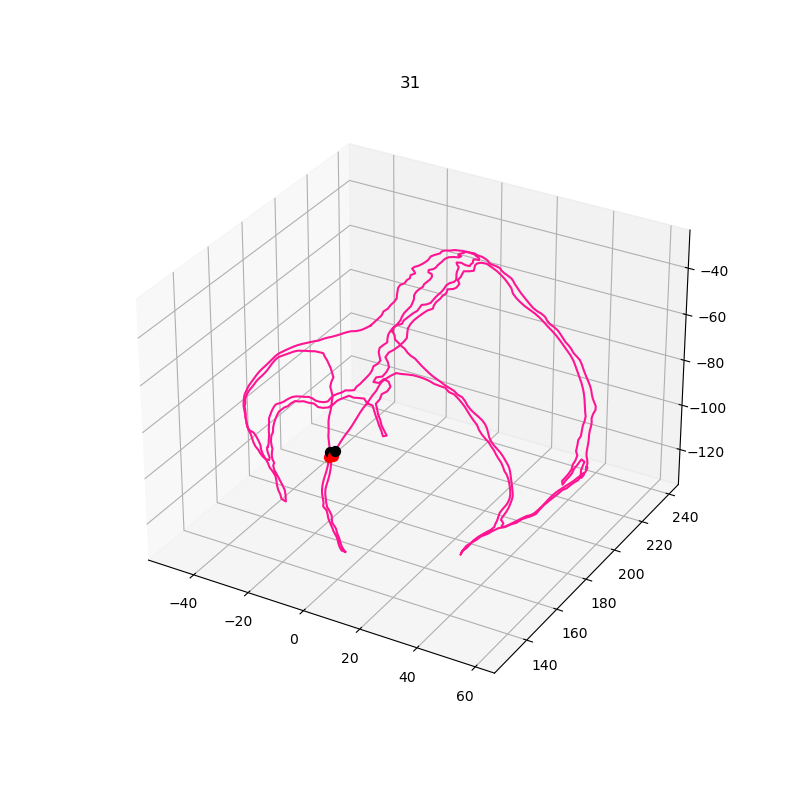

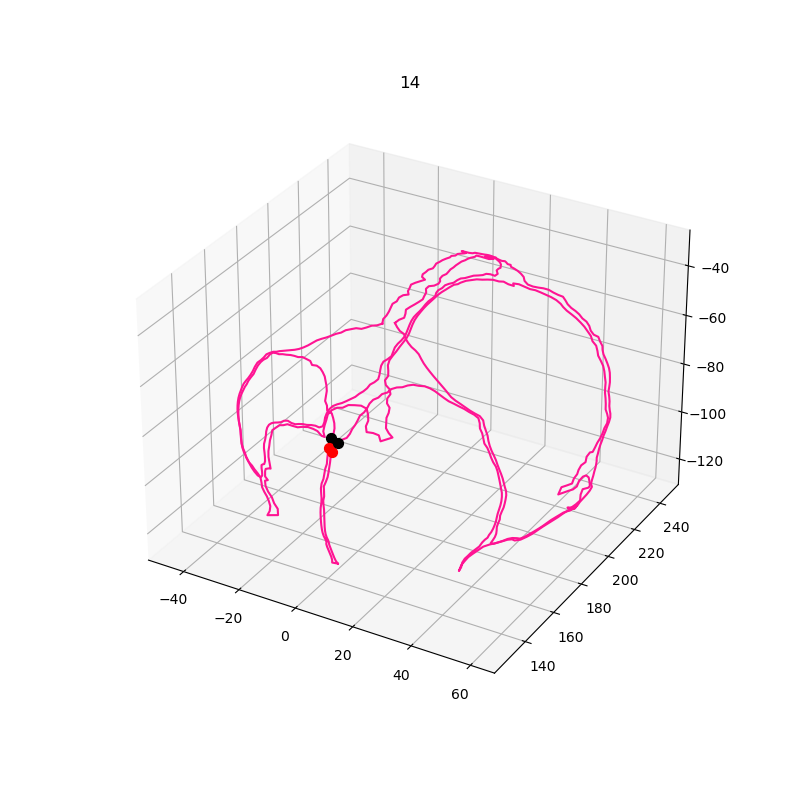

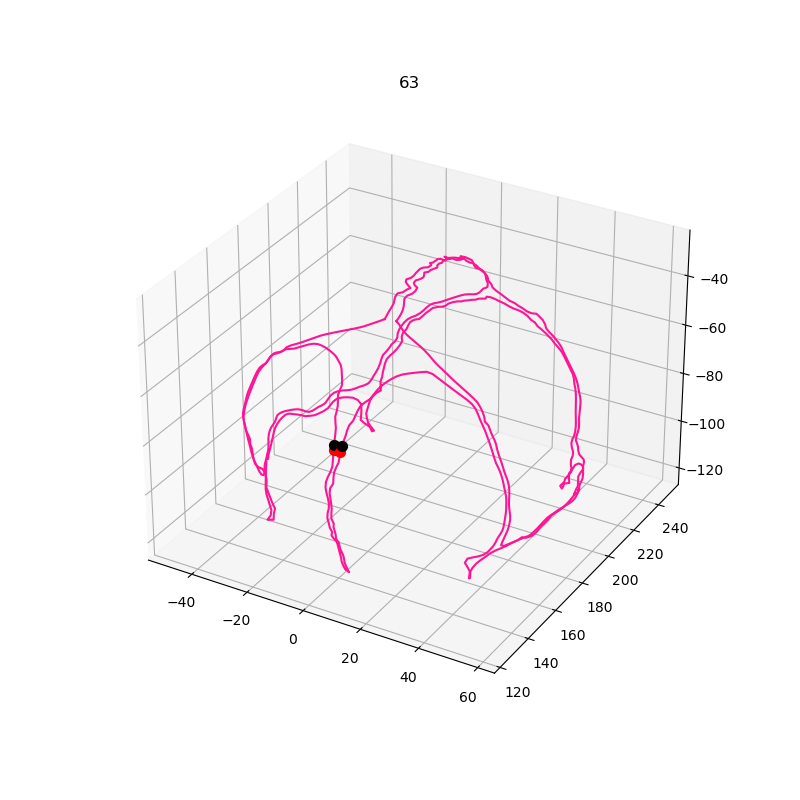


**Figure 2** The comparison of JP3 and JP4 under different *k* values (points of *k* =0.16 were shown in red, and point of *k*=0.15 were shown in black).

***k* of 0.18 for JP7 and JP8**

Similar to the metopic suture, for JP7 and JP8, smaller values of *k* resulted in identified sagittal suture having a larger width at the junction with posterior fontanelle, as shown in the figure below. It is considered that the black points were more representative of JP7 and JP8 compared to the red points. *k* values less than 0.17 of JP7 and JP8 yielded similar results.


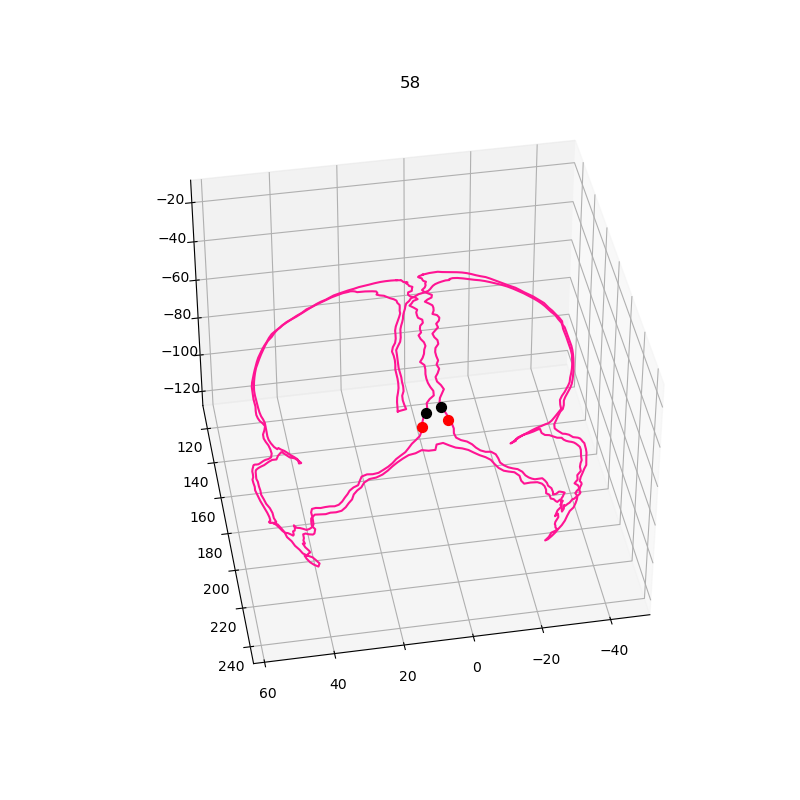

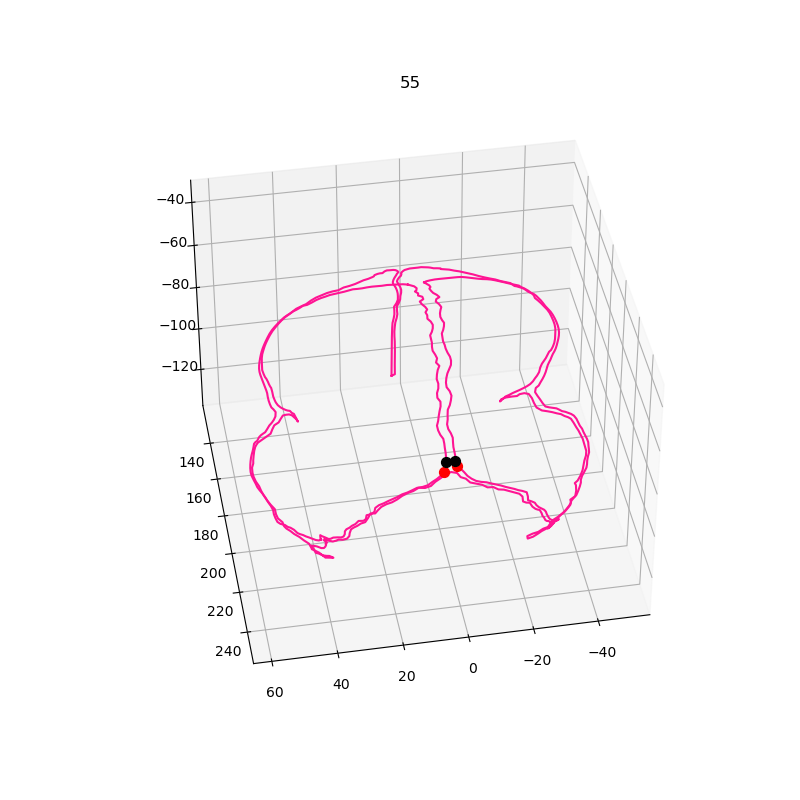

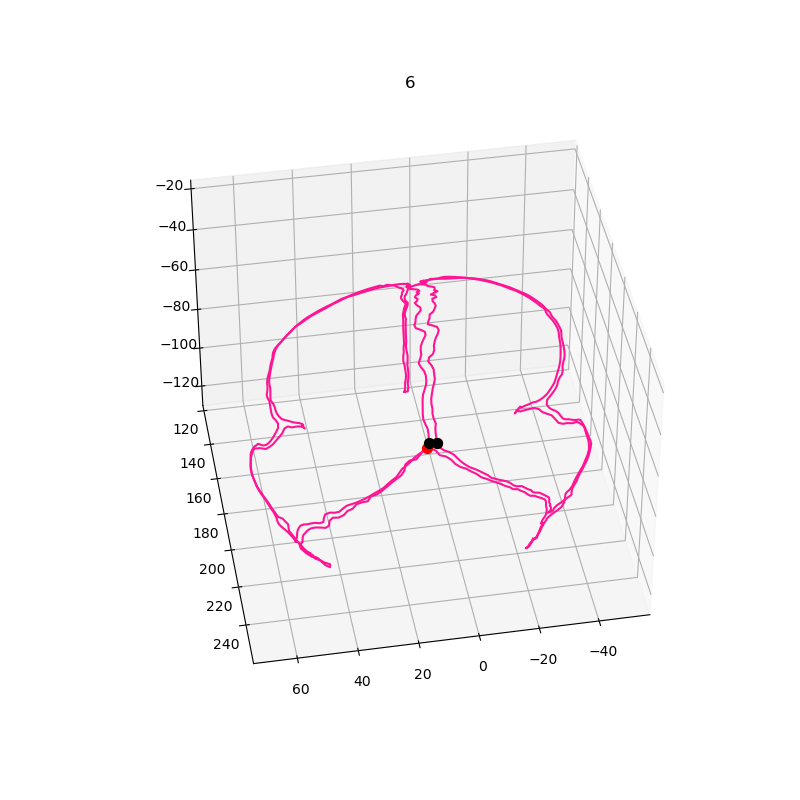

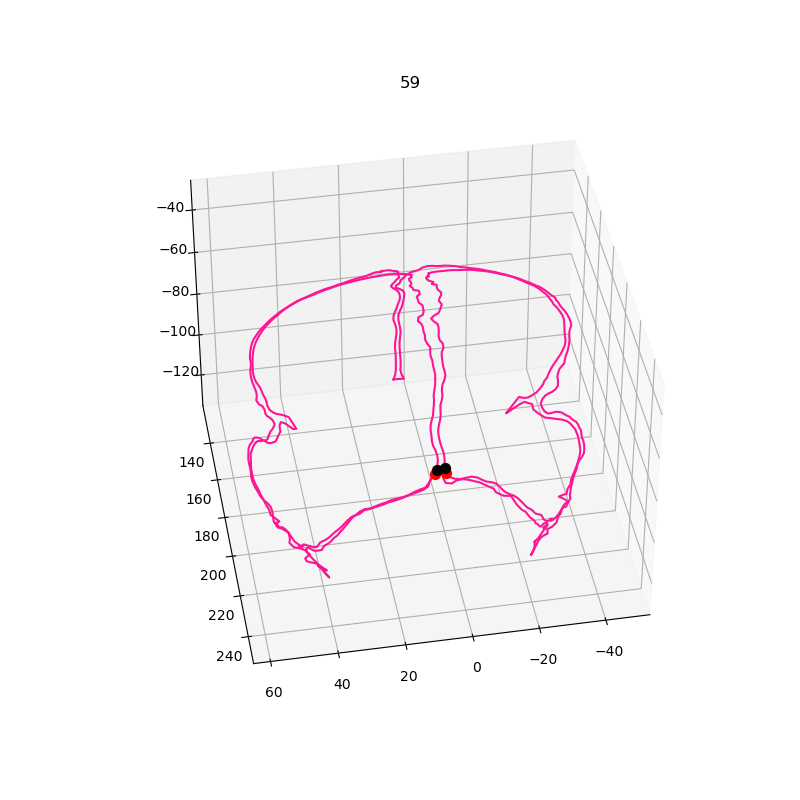


**Figure 3** The comparison of JP7 and JP8 under different *k* values (points of *k* =0.17 were shown in red, and point of *k*=0.18 were shown in black).

If k was set to 0.19 or another higher value, it would cause the positions of JP7 and JP8 to move further upwards towards the calvaria. Consequently, the recognized posterior fontanelle size became larger, resulting in an increased area.


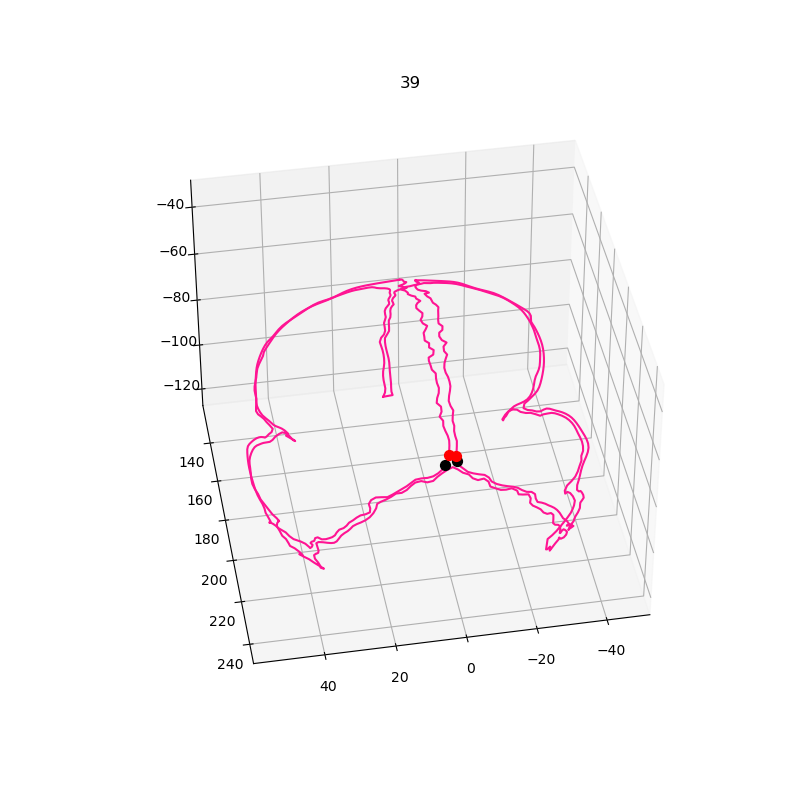

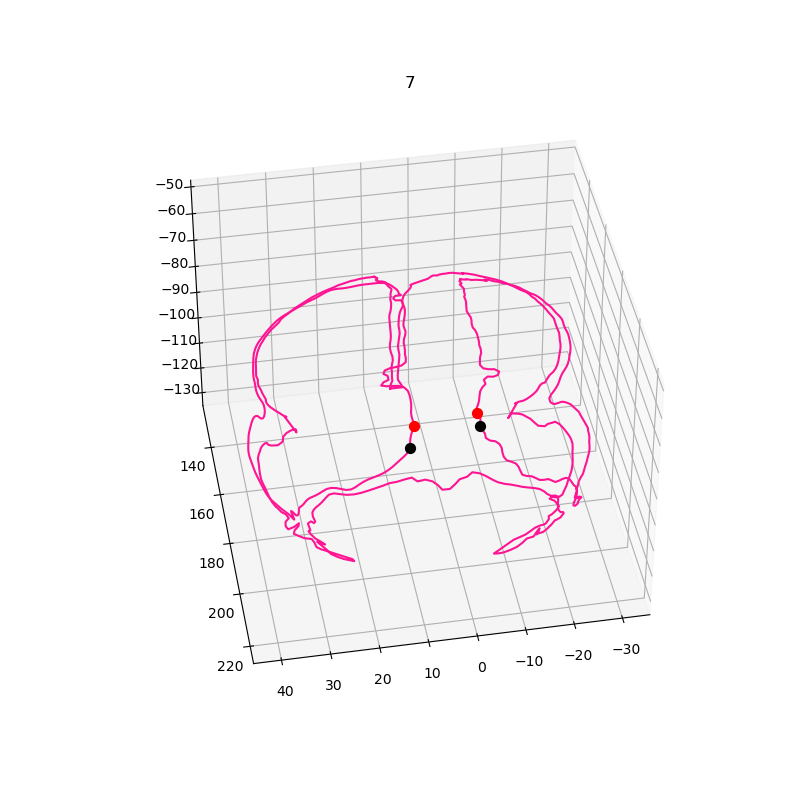

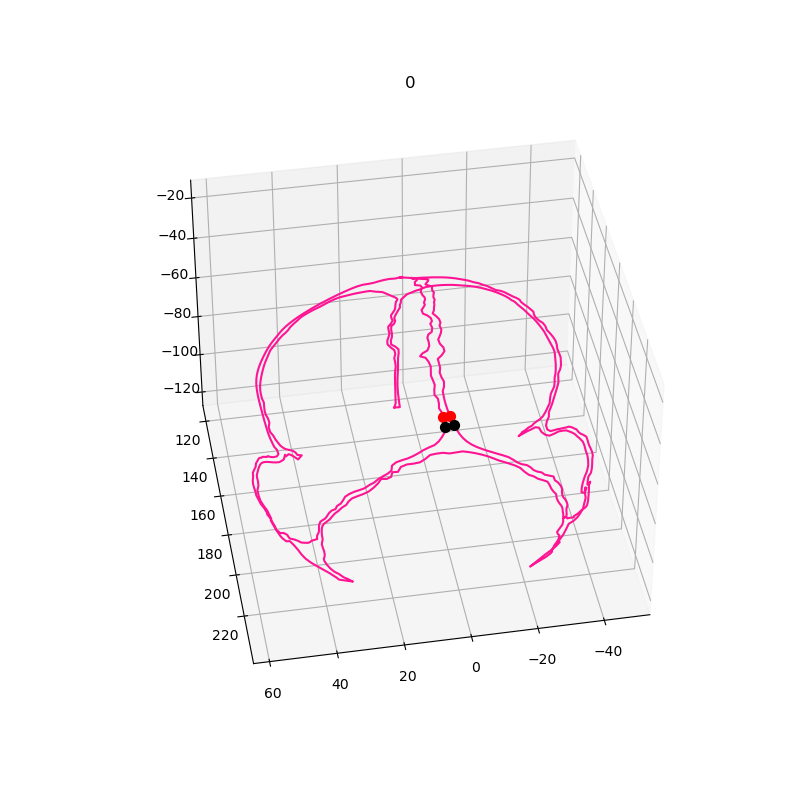

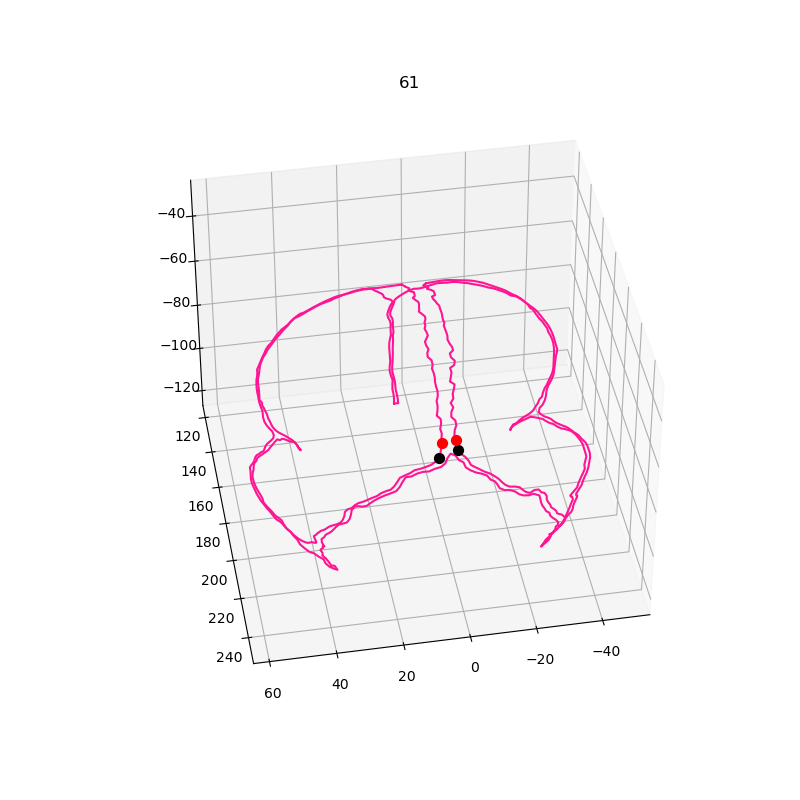


**Figure 4** The comparison of JP7 and JP8 under different *k* values (points of *k* =0.19 were shown in red, and point of *k*=0.18 were shown in black).

***m* of 0.4 for JP9-32 (The blue junction points in Figure 3)**

If m was set to 0.3 or another smaller value (taking an example of JP9-12), it would cause both the length and width of the left coronal suture to decrease.

**
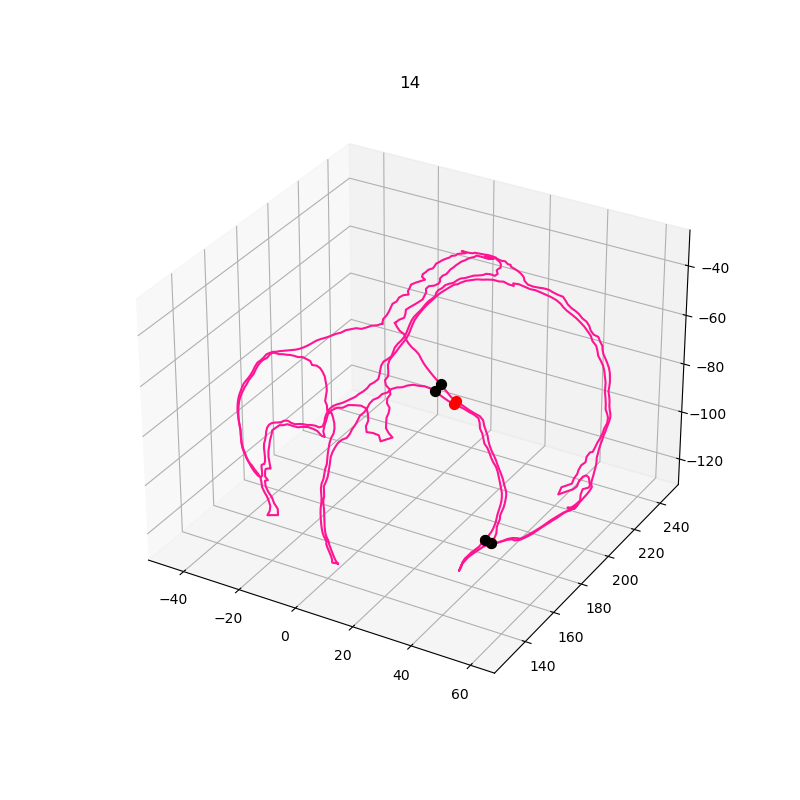

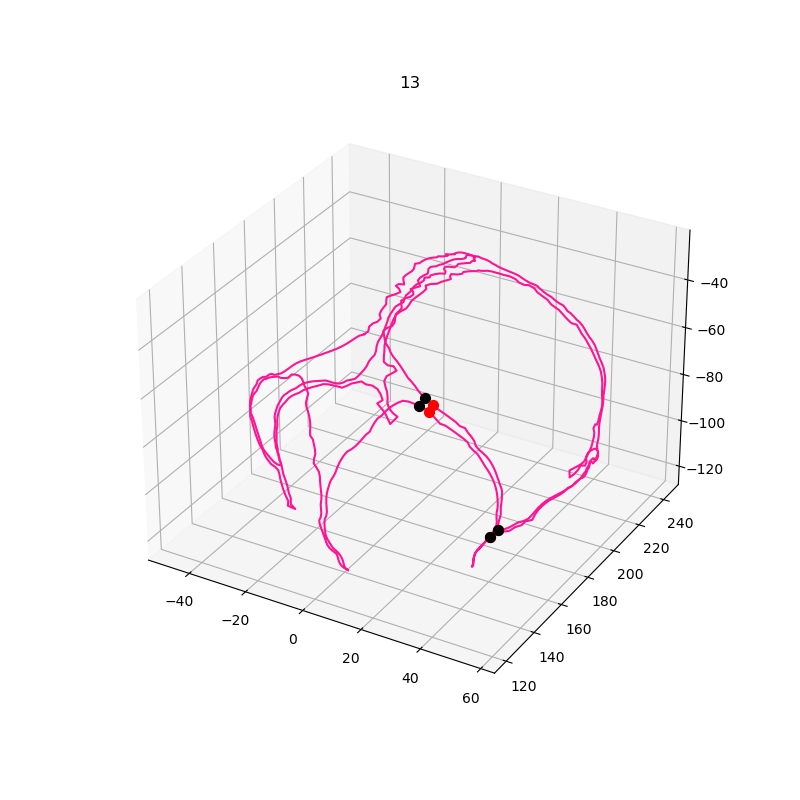

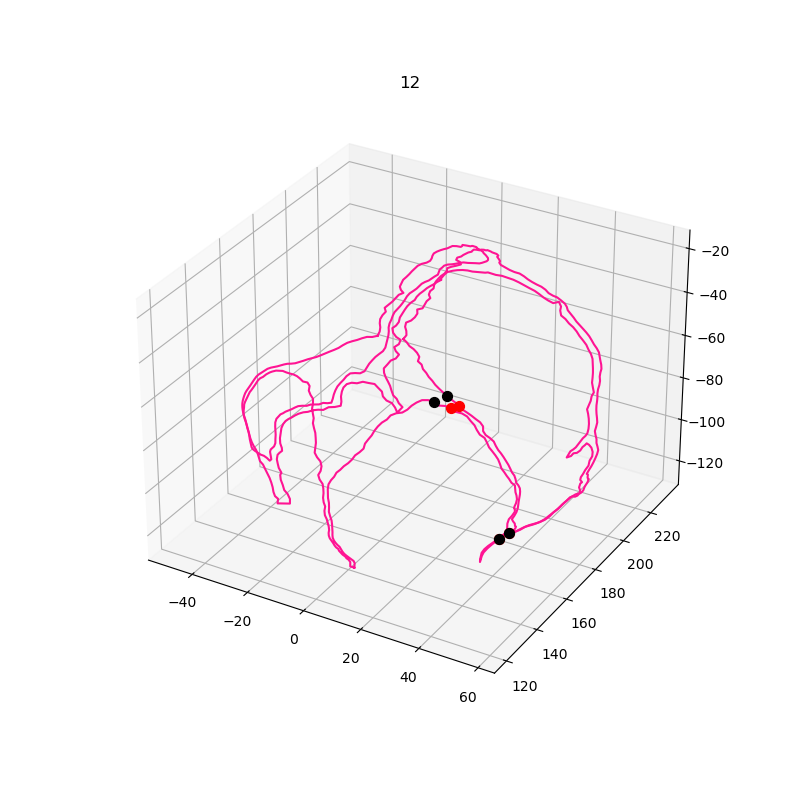

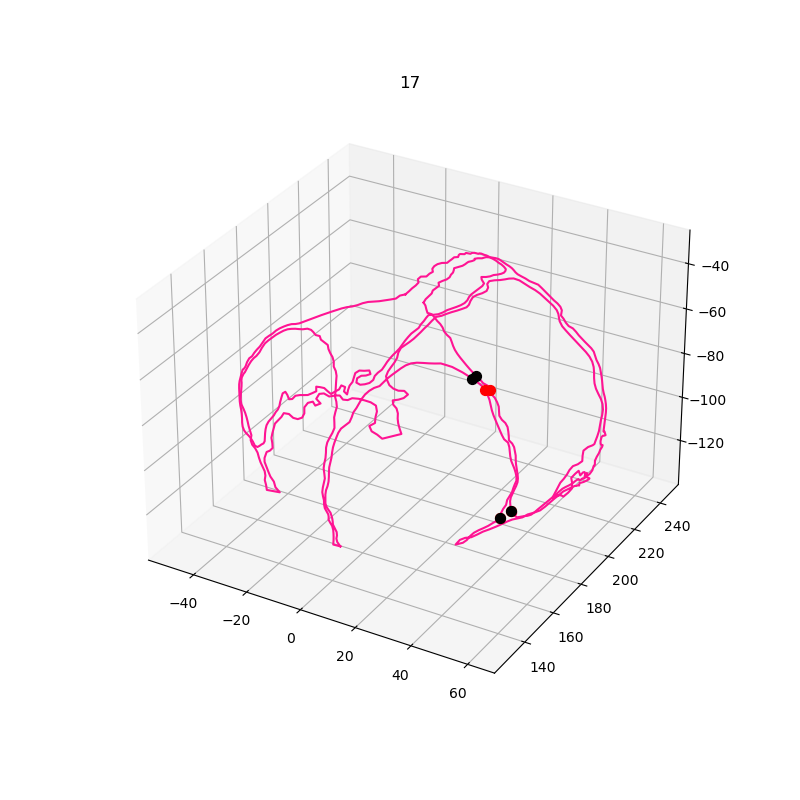
**

**Figure 5** The comparison of JP9 and JP10 under different *m* values (points of *m*=0.3 were shown in red, and point of *m*=0.4 were shown in black).

On the contrary, if m was set to 0.5, the measured anterior fontanlle size decreased, resulting in an increase in both the length and width of coronal sutures.


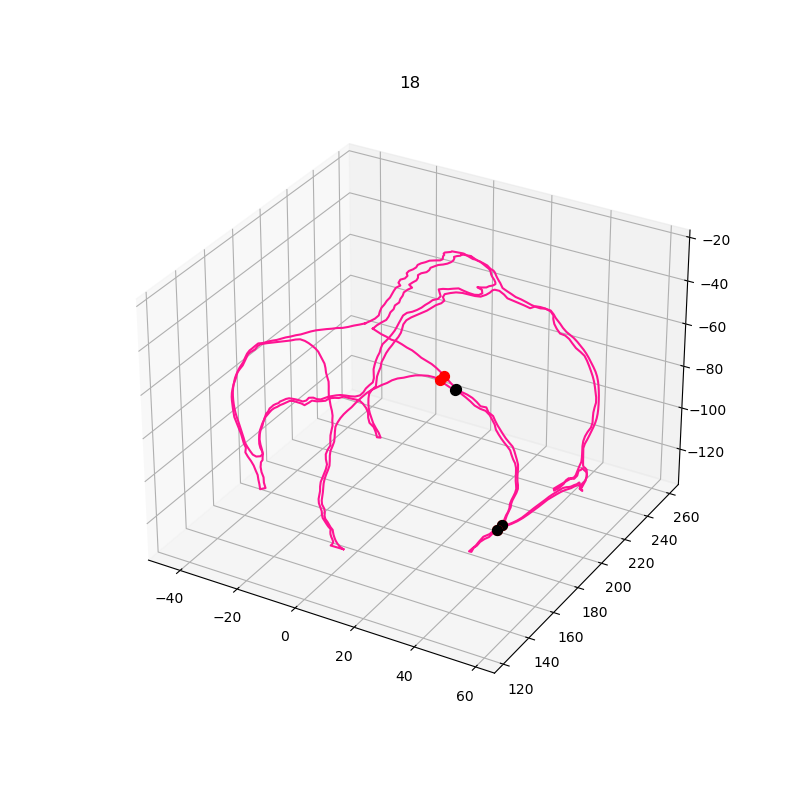

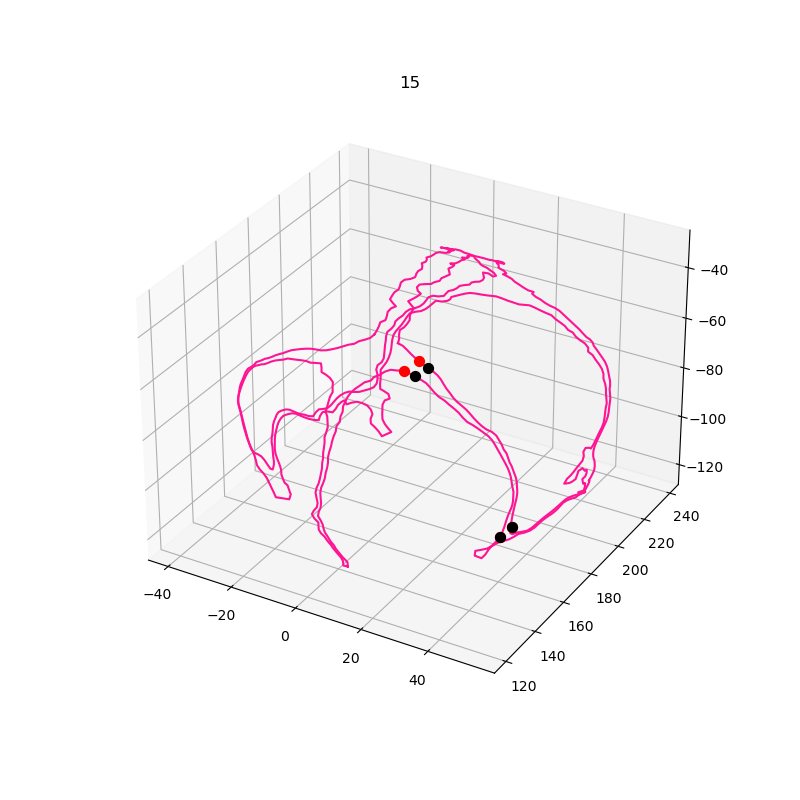

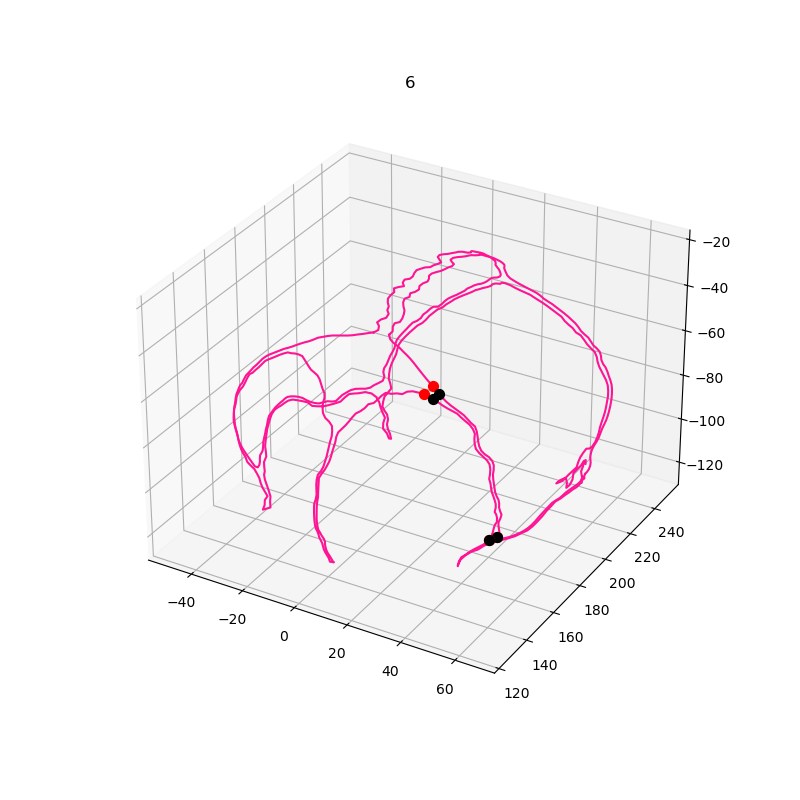

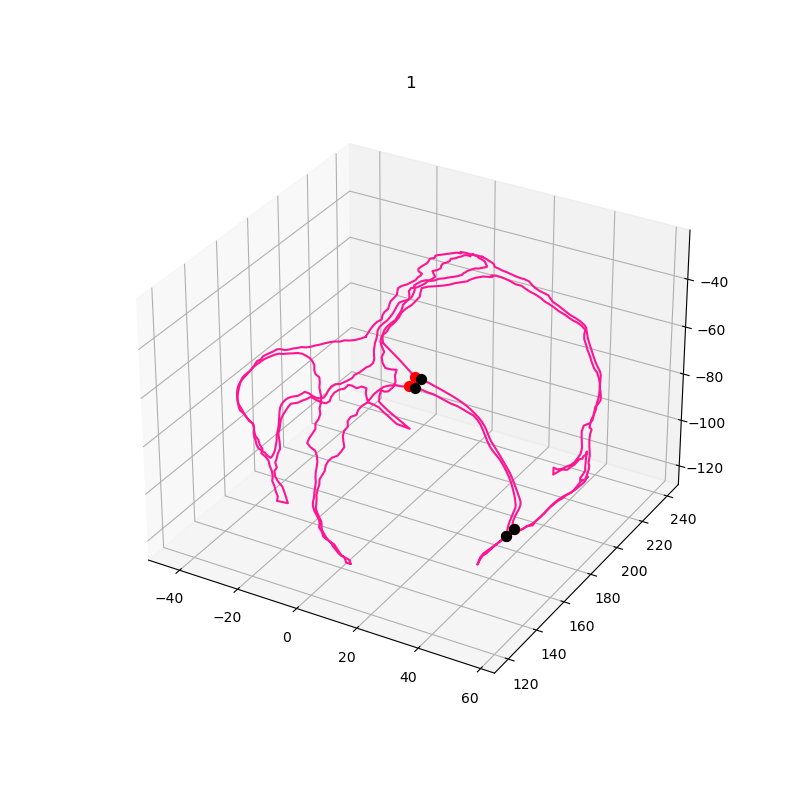


**Figure 6** The comparison of JP9 and JP10 under different *m* values (points of *m*=0.5 were shown in red, and point of *m*=0.4 were shown in black).
